# Efficacy and safety profile of intravitreal dexamethasone implant versus antivascular endothelial growth factor treatment in diabetic macular edema: a systematic review and meta-analysis

**DOI:** 10.1038/s41598-023-34673-z

**Published:** 2023-05-08

**Authors:** Sheng-Chu Chi, Yi-No Kang, Yi-Ming Huang

**Affiliations:** 1 Department of Ophthalmology, Faculty of Medicine, Taipei Veterans General Hospital, Taipei, Taiwan; 2 National Yang Ming Chiao Tung University, Taipei, Taiwan; 3 Evidence-Based Medicine Center, Wan Fang Hospital, Taipei Medical University, Taipei, Taiwan; 4 Research Center of Big Data and Meta-Analysis, Wan Fang Hospital, Taipei Medical University, Taipei, Taiwan; 5 Cochrane Taiwan, Taipei Medical University, Taipei, Taiwan; 6 Institute of Health Policy and Management, College of Public Health, National Taiwan University, Taipei, Taiwan

**Keywords:** Drug development, Endocrine system and metabolic diseases

## Abstract

To better understand the efficacy of intravitreal dexamethasone implant (Ozurdex) versus antivascular endothelial growth factor (anti-VEGF) treatment in patients with diabetic macular edema (DME). A systematic review and meta-analysis. The study included randomized control trials (RCTs) and non-randomized control trials (Non-RCTs) before December 2021 that compare the efficacy of Ozurdex-related therapyand anti-VEGF therapy. We searched PubMed, Cochrane Library, and EMBASE. The quality of the included studies was assessed carefully. 30 studies were included. Regarding BCVA change, the overall result revealed no significant differences between Ozurdex and anti-VEGF therapies in patients with nonresistant DME, but Ozurdex group had significantly more VA improvement than anti-VEGF therapies in patients with resistant DME (MD 0.12, 95% CI 0.02–0.21). In terms of central retinal thickness (CRT) decrease, there was a significant difference between Ozurdex therapy and anti-VEGF therapy in patients with nonresistant DME (MD 48.10, 95% CI 19.06–77.13) and resistant DME (MD 65.37, 95% CI 3.62–127.13). Overall, Ozurdex therapy resulted in significantly greater VA improvement and CRT decrease than anti-VEGF therapy in resistant DME patients. Ozurdex therapy was not inferior to anti-VEGF therapy in patients with nonresistant DME.

## Introduction

Diabetes is a chronic metabolic disease that leads to various microvascular and macrovascular complications. The number of people diagnosed as having diabetes mellitus has been estimated to increase to 700 million globally by the year 2045^1^. Diabetic macular edema (DME) is a vision-threatening complication in patients with diabetic retinopathy^2,3^. DME occurs secondary to retinal barrier rupture and is caused by changes in blood sugar levels resulting from hyperglycemia. Vascular endothelial growth factor (VEGF) plays a crucial role in the pathogenic process of DME. This pathogenic process further leads to the accumulation of extracellular fluid^2^. In recent years, emerging evidence has revealed that inflammation is involved in the pathogenesis of DME. Inflammations initiated by microglial cells, Müller cells, astrocytes, and several cytokines have been reported to be associated with the maintenance of low-grade inflammation^4–6^. Furthermore, a previous study revealed both VEGF and cytokine levels to be significantly higher in patients with hyperfluorescent DME than in those with inactive DME^7^. Anti-VEGF therapy has been developed based on the aforementioned pathogenesis of angiogenesis. Previous studies have revealed anti-VEGF therapy to be superior to laser therapy in treating DME^8–10^. Currently, the intravitreal injection of anti-VEGF remains the first-line therapy for DME. However, some patients do not exhibit a good response to anti-VEGF^11,12^. A post hoc analysis of a randomized controlled trial (RCT) assessing the cumulative 3-year probabilities of chronic persistent DME reported that 40.1% of eyes had chronic persistent DME^11^.

The use of corticosteroids is another potential therapeutic option, as corticosteroids decrease VEGF synthesis and pro-inflammatory cytokine concentrations^13^. The 0.7 mg intravitreal DEX implant Ozurdex® (Allergan, Inc.) was designed to deliver medication to the retina and could maintain the drug concentration in the vitreous body for up to 6 months^3^. Several research groups thereafter initiated head-to-head clinical trials to compare the efficacy of anti-VEGF therapy and Ozurdex therapy^14–58^. BEVORDEX, the first RCT to assess this, reported that Ozurdex therapy was not inferior to anti-VEGF therapy in terms of visual acuity (VA) changes and that it led to superior anatomical outcomes compared with anti-VEGF therapy at the primary endpoint^35^. However, other studies have reported heterogeneous results. The potential factors associated with the efficacy of Ozurdex and anti-VEGF therapies emerged as crucial challenges in DME treatment.

To the best of our knowledge, no synthetic evidence exists regarding the possible confounding factors affecting the efficacy of anti-VEGF and Ozurdex therapies. Therefore, this systematic review and meta-analysis aimed to quantify the effects of these two therapies. Moreover, meta-regression was performed to determine the possible confounding factors when comparing these therapies.

## Research design and methods

This study was conducted in accordance with the PRISMA guidelines. As published data were used, our study was exempt from institutional review board approval. Our systematic review and meta-analysis aimed to assess the efficacy of anti-VEGF and Ozurdex therapies in treating DME and to determine the potential confounding factors affecting the therapeutic efficacy. The study was registered on Research Registry (Review Registry UIN:reviewregistry1422).

### Search strategy and study selection

Our meta-analysis included all RCTs and observational studies that met the following criteria: (1) the study recruited patients with DME and (2) the intervention consisted of anti-VEGF therapy and Ozurdex therapy. Studies that included patients with not only DME but also other specific ocular diseases, such as concurrent epiretinal membrane and gray literature that did not report details, were excluded. Some studies included specific patients with resistant DME, while others recruited patients with nonresistant DME. Most studies define the resistant DME as the DME with suboptimal treating response after three monthly anti-VEGF injections. A meta-analysis of these two groups was performed in our study. While Ozurdex monotherapy was used in some studies, combination therapy was used in others. A subgroup analysis was further performed in our study to assess the treatment modality.

**Figure 1:**
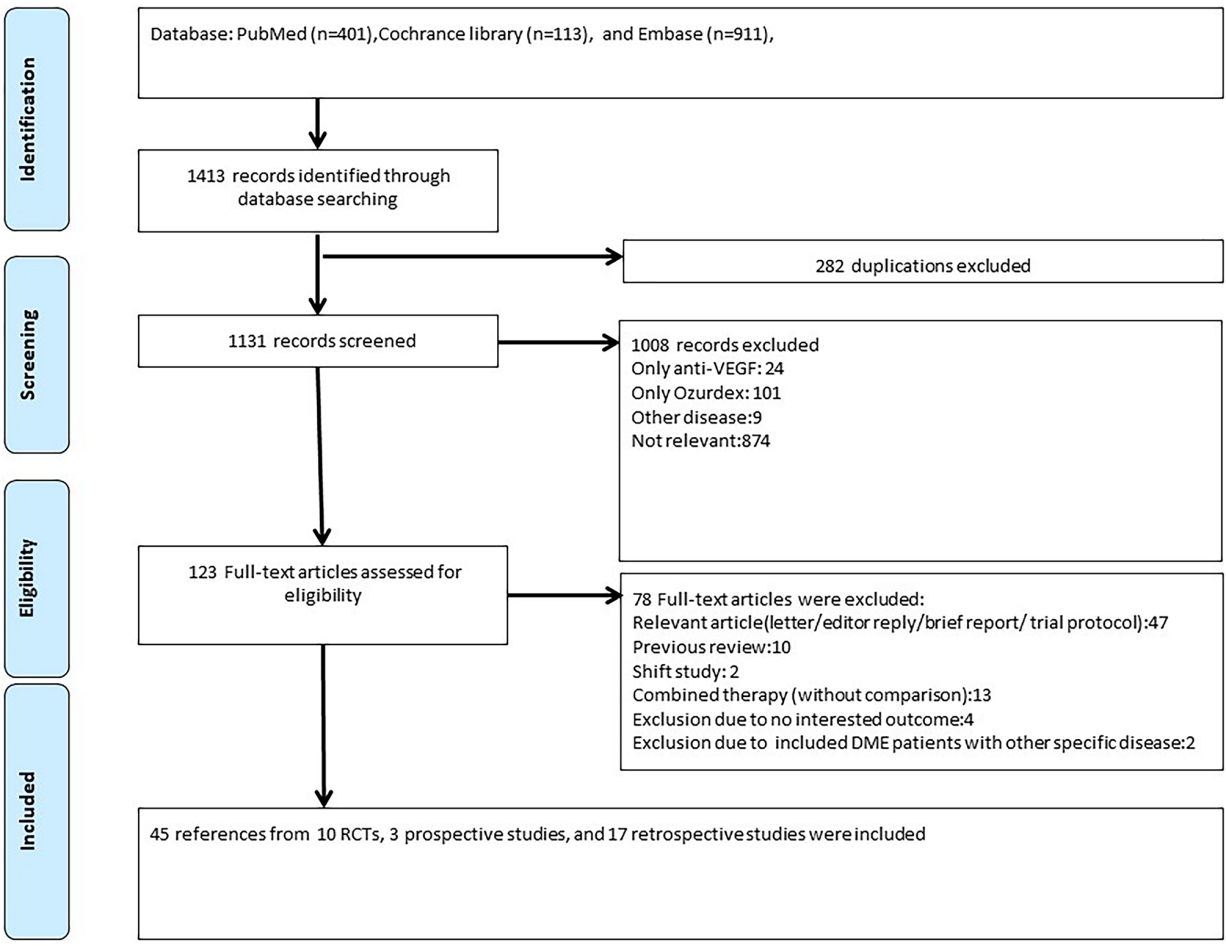
Flowchart of study selection.

PubMed, Embase, and the Cochrane Library were searched for studies published before April 2021 without any language limitation. The search strategy consisted of the relevant terms anti-VEGF, Ozurdex, and DME in free text and Medical Subject Headings. The details of the search strategy are listed in Supplementary. The titles and abstracts were independently screened by two reviewers (SCC and YNK) after the search. Duplications and irrelevant references were removed, and full texts were then retrieved for further review (Fig. 1).

### Quality assessment

Two reviewers (SCC and YNK) independently assessed the risk of bias of the included RCTs by using the Cochrane Risk of Bias tool. Non-RCTs are assessed using the Risk of Bias In Non-randomized Studies—of Interventions (ROBINS-I) tool. If any disagreement occurred regarding the quality assessment, it was resolved through discussion with another author (HYM).

### Data extraction

The following information was extracted by two independent reviewers (SCC and YNK): study year; study population; study location; and data, including the change in VA, decrease in central retinal thickness (CRT), safety outcomes (including intraocular pressure [IOP] change), and occurrence of severe ocular adverse events. The log minimum angle of resolution (logMAR) scale was used in this study. The Early Treatment Diabetic Retinopathy Study (ETDRS) letter scores, Snellen chart scores, and decimal acuity scores were converted to logMAR by using a previously reported conversion formula^59^.

Only one anti-VEGF therapy arm was assessed if a study included two treatment arms using different anti-VEGF agents. Under the circumstances, we evaluated the treatment arm, which included more patients. The mean and standard deviation (SD) data for continuous outcomes and the event and total sample size data for binary outcomes were extracted. Regarding the outcome of VA improvement, relevant SD data could not be extracted from the original article of Callanan et al. Therefore, the data of the study was not pooled in the analysis. Moreover, the change in VA reported using Snellen chart scores in the study of Aksoy et al. could not be converted to logMAR. Therefore, the outcome of VA improvement in the study of Aksoy et al. was not included in our analysis.

### Statistical methods and data synthesis

Quantitative data synthesis was performed using pairwise meta-analyses. A random-effects model was used in our meta-analysis because of conceptual heterogeneity. The mean differences (MDs) with 95% confidence intervals (CIs) were calculated for continuous outcomes, and the risk ratios (RRs) were calculated for binary outcomes. The I^2^ statistic was used for assessing statistical heterogeneity. An outcome was defined as highly heterogeneous if I^2^ was higher than 50%. Funnel plots and Egger’s test were used to assess the small-study effect. For determining potential factors that could affect the pooling results, subgroup analysis and meta-regression were performed to assess the effects in patients with nonresistant DME. In the meta-regression, the mean age, treatment duration, baseline best-corrected visual acuity (BCVA), baseline CRT, glycated hemoglobin (HbA1C) level, and lens status served as predictors. All statistical analyses were performed using R software (4.2.0).

## Results

A total of 1,413 potential references were obtained from PubMed (n = 401), Embase (n = 911), and the Cochrane Library (n = 113). After the exclusion of 282 duplicate references, the remaining articles were assessed for eligibility. Finally, 21 articles from 10 RCTs, 3 prospective non-RCTs, and 21 articles from 17 retrospective studies were included in this meta-analysis (Fig. 1). No evidence of asymmetry was noted by visual inspection in all funnel plots. (Supplementary-2, 3, 4, 5).

### Study characteristics

A total of 2,409 eyes were included in our study. The mean age of the patients was 62.19 years old (range 56.2–67.8 years old). The follow-up duration ranged from 1 to 12 months. The baseline BCVA ranged from 0.2 to 0.96 logMAR, and the baseline CRT ranged from 261.1 to 605.3 µm. Five studies included patients with resistant DME, while 25 studies included patients with non-resistant DME. Eight of these studies included patients with naïve DME. Details regarding the characteristics of the included studies are summarized in Table 1.

**Table 1:** Characteristics of studies.

Study	Study type	Year	Follow up time(m)	Persisted DME Not persisted DME Naïve DME	Treatment	Number (eye)	Age (mean)	Gender (M/F)	Pseudophakia/phakia	A1C	BCVA (logMAR) (mean)	CRT (mean)
Aksoy et al.^26^	Retrospective	2020	3	Persisted DME	1. Aflibercept	34	59.3	18/16	6/31	N/A	0.74	529.1
					2. Ozurdex	37	61.3	1819	5/23		0.72	532.04
Aydin et al.^27^	Retrospective	2020	12	Not persisted DME	1. Ranibizumab	30	60.2	15/15	N/A	7.15	0.75	466.1
					2. Ozurdex	22	58.2	9/13		7.32	0.83	524.2
Bolubasi et al.^21^	Retrospective	2019	3	Not persisted DME	1. Bevacizumab	32	56.4	13/19	9/23	7.8	0.6	490.3
					2. Ozurdex	25	65.1	9/25	15/10	7.8	0.8	540
Busch et al.^22^	Retrospective	2019	12	Persisted DME	1. Anti-VEGF	38	61.2	17/21	15/23	N/A	0.54	496.2
					2. Ozurdex	38	63.1	22/16	18/20		0.54	504.7
Callanan et al.^41^	RCT	2017	12	Not persisted DME	1. Bevacizumab	182	63.7	116/62	62/120	N/A	0.49	471
					2. Ozurdex	181	63.4	112/69	54/127		0.50	465
Ceravolo et al.^28^	Retrospective	2020	3/2	Naïve DME	1. Ranibizumab	75	70.8	110/47	N/A	N/A	0.67	261.1
					2. Ozurdex	81	70.4	112/49			0.74	271.5
Comet et al.^56^	Prospective	2020	12	Naïve DME	1. Ranibizumab	19	65.6	8/11	9/10	7.5	0.45	450.1
					2. Aflibercept	20	69.6	11/9	8/12	7.8	0.56	469.5
					3. Ozurdex	21	66.3	12/9	9/12	7.6	0.52	464.5
Demircan et al.^19^	Retrospective	2018	3	Not persisted DME	1. Ranibizumab	101	58	50/51	N/A	N/A	0.8	605.3
					2. Ozurdex	35	60.3	19/16			1.0	643.9
Giles et al. (BEVODEX)^35^	RCT	2014	12	Not persisted DME	1. Bevacizumab	42	62.2	26/16	N/A	7.8	0.57	503
					2. Ozurdex	46	61.4	30/16		7.7	0.59	474.3
Hernández-Bel et al.^23^	Retrospective	2019	12	Not persisted DME	1. Aflibercept	15	69.4	N/A	3/12	7.6	0.28	411
					2. Ozurdex + Aflibercept	15	66.2	N/A	4/11	8.2	0.19	411.4
Karakurt et al.^20^	Retrospective	2018	1	Not persisted DME	1. Ranibizumab	81	62.94	40/41	N/A	N/A	N/A	N/A
					2. Aflibercept	53	65.70	30/23				
					3. Ozurdex	54	59.47	36/18				
Kaya et al.^54^	RCT	2021	12	Naïve DME	1. Ranibizumab	34	66.2	12/12	10/24	7.0	0.66	Total
					2. Ozurdex + Ranibizumab	34	64.6	12/10	12/22	7.0	0.74	597
Limon et al.^57^	Prospective	2021	3	Persisted DME	1. Bevacizumab	30	63.20	14/16	30/0	N/A	0.83	434.42
					2. Ozurdex + Bevacizumab	35	64.34	12/17	31/4		0.82	462.34
Lin et al.^32^	Retrospective	2021	6	Not persisted DME	1.Aflibercept	50	63.4	26/14	16/34	7.4	0.63	420.6
					2. Ozurdex + Aflibercept	52	65.1	20/21	29/23	7.5	0.64	433.8
Maturi et al.^36^	RCT	2015	12	Not persisted DME	1. Bevacizumab	19	61	13/17	7/12	N/A	0.42	412
					2. Ozurdex + Bevacizumab	21	Total	Total	8/13		0.4	372
Maturi et al.^44^	RCT	2018	6	Not persisted DME	1. Ranibizumab	64	66*	28/36	32/32	7.4*	0.44	396
					2. Ozurdex + Ranibizumab	63	64*	34/31	26/39	7.1*	0.44	375
Mastropasqua et al.^24^	Retrospective	2019	6	Naïve DME	1. Ranibizumab	25	61.4	13/12	13/12	N/A	0.4	460.3
					2. Ozurdex	25	62.1	10/15	14/11		0.5	479.1
Mishira et al.^55^	RCT	2021	4	Not persisted DME	1. Ranibizumab	70	63.15	38/32	N/A	N/A	0.96	460
					2. Ozurdex	70	63.3	37/33			0.96	471
Muftuoglu et al.^33^	retrospective	2021	3	Not persisted DME	1. Ranibizumab	18	62.22	13/5	7/11	6.72	0.27	509
					2. Ozurdex	19	61.84	19/10	7/12	7.26	0.34	533
Ozsaygili et al.^52^	RCT	2020	12	Naïve DME	1.Aflibercept	50	66.4	15/14	23/27	8.2	0.75*	576.5*
					2.Ozurdex	48	64.8	20/13	19/29	8.4	0.77*	615.2*
Podkowinski et al.^49^	RCT	2019	5	Not persisted DME	1. Ranibizumab	9	66.89	N/A	N/A	N/A	0.2	440.89
					2.Ozurdex	9	64.56				0.36	471.33
Routier et al.^34^	Retrospective	2021	2	Naïve DME	1. Anti-VEGF	55	Total 65.9	Total 19/26	26/39	N/A	0.56	498
					2.Ozurdex	10					0.56	451
Rübsam et al.^58^	Prospective	2021	1	Not persisted DME	1. Anti-VEGF	40	Total 58.9	Total 39/12	N/A	N/A	total	455.5
					2.Ozurdex	19					0.41	471.6
Sever et al.^25^	Retrospective	2017	12	Not persisted DME	1. Ranibizumab	44	Total 56.2	N/A	5/39	N/A	0.75	372.6
					2.Ozurdex	40			10/30		0.75	376.6
Shah et al.^40^	RCT	2016	7	Not persisted DME	1.Bevacizumab	23	61	13/10	9/14	N/A	0.52	485
					2.Ozurdex	27	65	12/15	14/13		0.52	458
Sharma et al.^50^	RCT	2019	6	Persisted DME	1.Anti-VEGF	20	56.42	N/A	0/45	N/A	0.51	460.95
					2.Ozurdex	20	58.15				0.56	452.25
Shin et al.^17^	Retrospective	2017	3	Not persisted DME	1.Bevacizumab	25	59.1	16/9	7/4	N/A	0.63	451.3
					2.Ozurdex	20	61.5	10/10	8/3		0.78	510.3
Thomas et al.^15^	Retrospective	2016	3	Persisted DME	1. Ranibizumab	11	Total 62	Total 4/7	4/7	Total 6.51	0.39	421.1
					2.Ozurdex	11			7/4		0.42	461.3
Vujosevic et al.^18^	Retrospective	2017	1	Naïve DME	1. Ranibizumab	26	65.2	15/11	N/A	7.5	0.55	518
					2.Ozurdex	23	66.9	15/8		7.3	0.68	594.6
Vujosevic et al.^30^	Retrospective	2020	4/3	Naïve DME	1. Ranibizumab	18	63.8	N/A	N/A	8.4	0.36	420.48
					2.Ozurdex	15	63.3			8.7	0.49	565.5

*Median

*DME* diabetic macular edema.

### BCVA change

**Figure 2:**
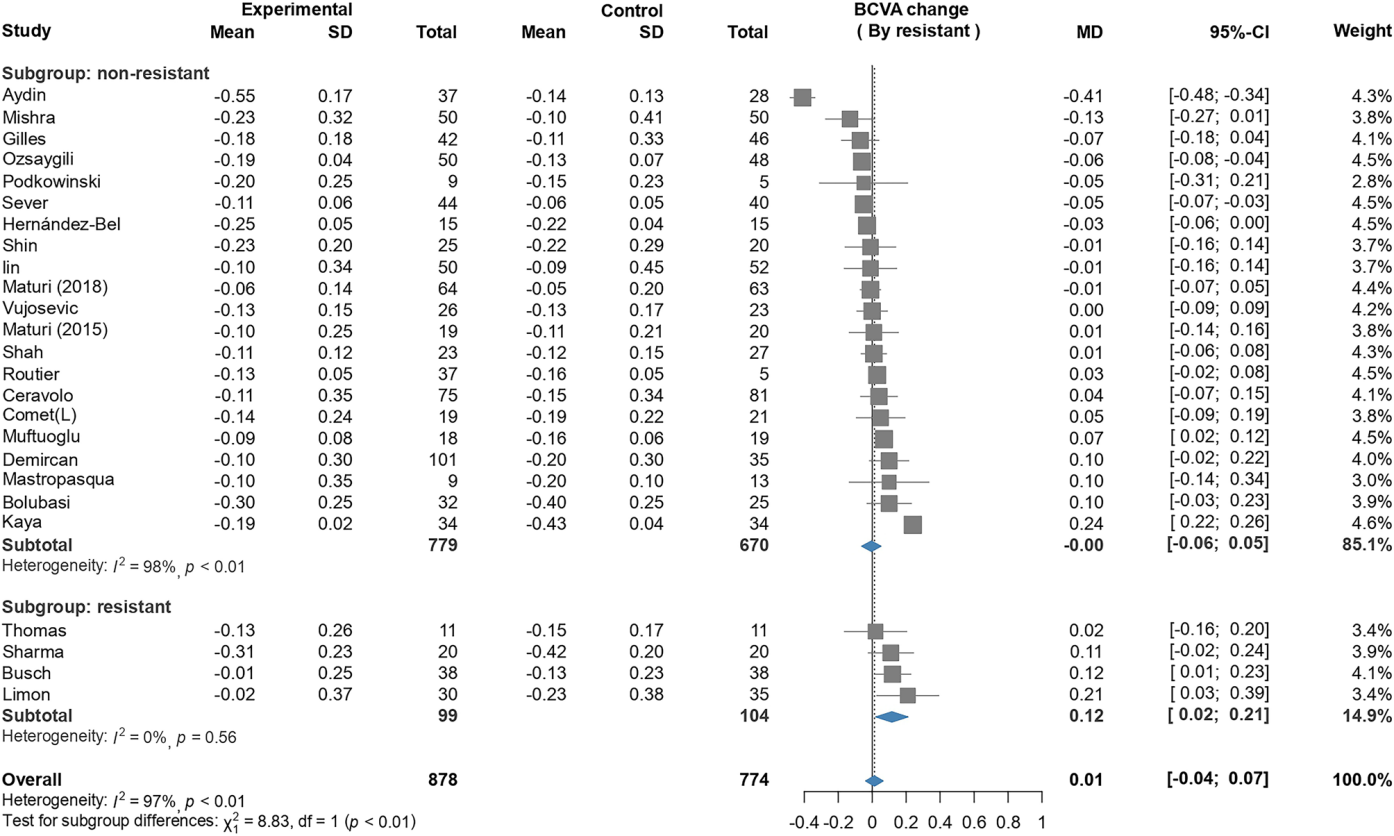
Forest plot of BCVA change.

Overall, 27 trials reported the outcome of BCVA change. 4 of these trials included patients with resistant DME. The pooled results revealed no significant differences between Ozurdex group and anti-VEGF group, with high heterogeneity being noted in patients with nonresistant DME (MD 0.00, 95% CI − 0.06–0.05, I^2^ = 97%, Figs. 1 and 2). However, in patients with resistant DME, a significant difference was noted between Ozurdex group and anti-VEGF group (MD 0.12, 95% CI 0.02–0.21, I^2^ = 0%, Fig. 2). Subgroup analysis of the treatment modality in patients with nonresistant DME revealed no significant difference between Ozurdex therapy and anti-VEGF therapy. Moreover, no significant difference was noted between anti-VEGF therapy and combined anti-VEGF + Ozurdex therapy (Supplementary-6). In both linear and nonlinear meta-regression models, the mean age, treatment duration, baseline BCVA, baseline CRT, and HbA1C level were not associated with the MDin BCVA change between the Ozurdex and anti-VEGF groups in patients with nonresistant DME. However, the MD in CRT change was significantly associated with the MD in BCVA change in the nonlinear meta-regression model (Supplementary-7). Furthermore, no significant association was noted between the MD in BCVA change and lens status. Although a significant association was noted between the MD in BCVA change and the proportion of patients with pseudophakia (P = 0.05, deviance = 6.67, Supplementary-7), this analysis was not compatible with the clinical status.

### CRT decrease

**Figure 3:**
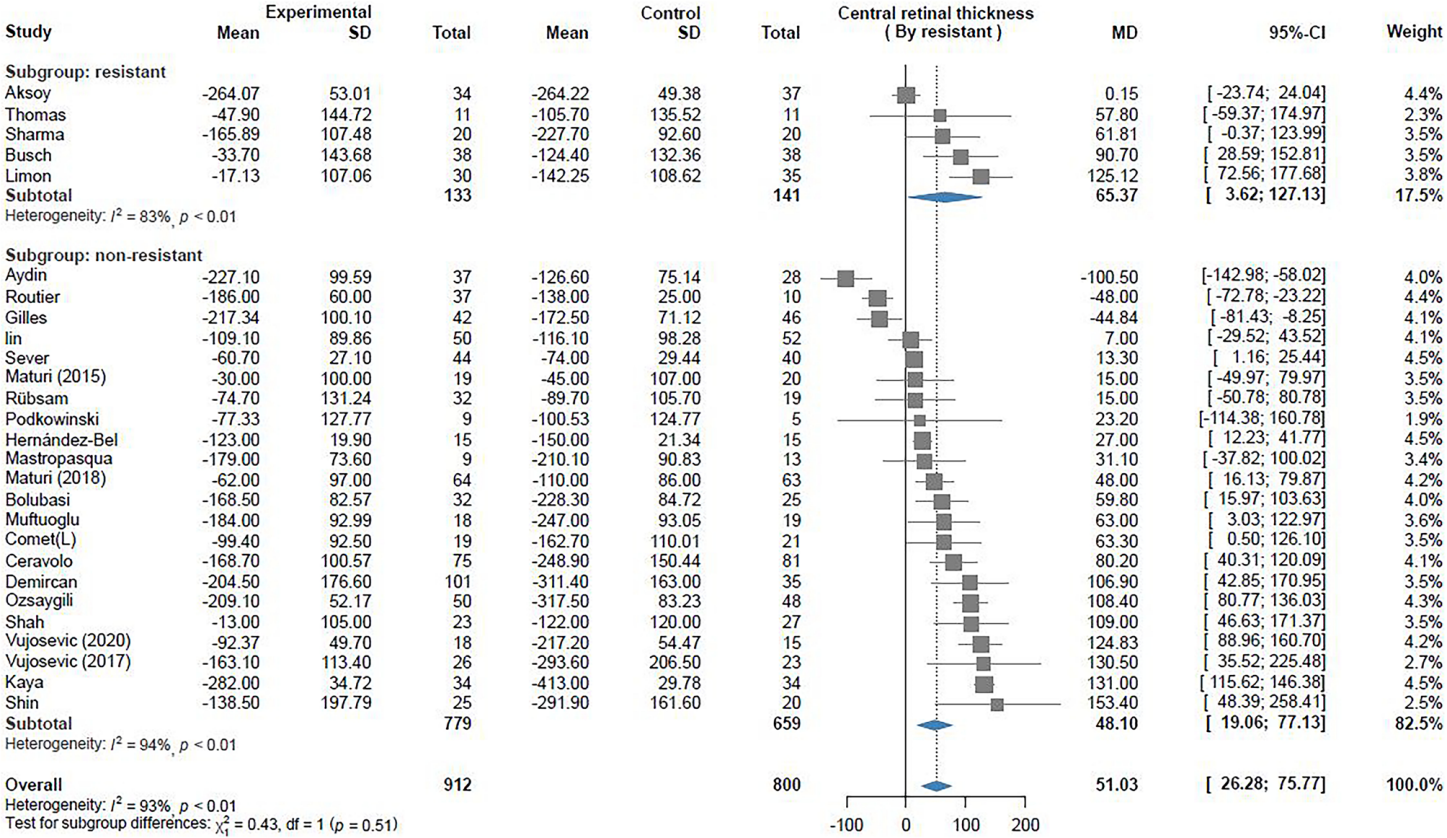
Forest plot of CRT change.

28 trials reported the outcome of CRT decrease. 5 of these trials included patients with resistant DME. Pooled analysis of patients with resistant DME revealed a greater decrease in CRT in the Ozurdex group than in the anti-VEGF group (MD 65.37, 95% CI 3.62 –127.13, I^2^ = 83%, Fig. 3); however, high heterogenicity was noted. Moreover, analysis of patients with nonresistant DME revealed a significant difference between Ozurdex group and anti-VEGF group (WMD 48.10, 95% CI 19.06–77.13, I^2^ = 94%, Fig. 3). Subgroup analysis revealed a significant difference between Ozurdex therapy and anti-VEGF therapy. Although a combination of Ozurdex and anti-VEGF could not lead to a significantly greater decrease in CRT than either therapy alone, a trend favoring the combination therapy in terms of CRT decrease was noted (Supplementary-8). The results of meta-regression revealed no association between CRT decrease and the mean age, treatment duration, baseline BCVA, baseline CRT, and HbA1C level. However, in the nonlinear meta-regression model, a significant association was noted between the MD in CRT decrease and baseline CRT. The parabola in the meta-regression revealed that Ozurdex therapy led to a greater decrease in CRT if the baseline CRT was more than 410um (P = 0.025, deviance = 130.27, Supplementary-9).

### IOP change

A total of 10 trials reported the outcome of IOP change. Only one of these trials included patients with resistant DME. The pooled results revealed no significant difference in IOP change between Ozurdex group and anti-VEGF group (MD − 0.42, 95% CI − 1.00–0.15, I^2^ = 40%, Supplementary-10). Moreover, subgroup analysis of the treatment modality in patients with nonresistant DME revealed that Ozurdex therapy did not lead to a significant increase in IOP. (Supplementary-10) Meta-regression could not detect the association between IOP change and the baseline IOP, mean age, and treatment duration (Supplementary-11).

### Severe ocular adverse events

Overall, 13 trials reported the outcome of the occurrence of severe ocular adverse events. 3 of these trials included patients with resistant DME. The severe ocular adverse events reported in these trials included endophthalmitis, retinal detachment, and glaucoma that could not be controlled by glaucomatous eye drops. The pooled results revealed no significant difference between Ozurdex group and anti-VEGF group, with low heterogeneity being noted (RR 0.64, 95% CI 0.28–1.49, P = 0.63, I^2^ = 0% Supplementary-12).

### Risk of bias assessment

Generally, the quality of the 9 included RCTs was moderate, with some “unclear” and “high risk”. “Some concerns” risk of bias was raised in the domain of random sequence generation and allocation concealment due to the incomplete information in the article. “High risk” in the blinding domain was raised in some studies due to the studies were unmasked. The observational studies were assessed and qualified by (ROBINS-I) tool. Overall, the level of evidence of included studies was downgraded due to the high risk of bias of confounding factors and the moderate risk of bias in the measurement of the outcomes. The detail of the risk of bias assessment was listed in the Supplement (Supplementary-13, 14).

## Discussion

### Summary of findings

This is the systematic review and meta-analysis with meta-regression on DME treatment. 30 studies published before April 2021 were included. The overall results revealed no significant differences between Ozurdex and anti-VEGF therapies in terms of BCVA change in patients with nonresistant DME. However, in patients with resistant DME, Ozurdex therapy led to significantly greater VA improvements than anti-VEGF therapy. Regarding CRT decrease, a significant difference was noted between Ozurdex therapy and anti-VEGF therapy in patients with nonresistant DME as well as those with resistant DME. Subgroup analysis of the treatment modality of Ozurdex (combination therapy or monotherapy) did not reveal any reduction in the heterogeneity of BCVA change and CRT decrease.No significant difference was noted between Ozurdex therapy and anti-VEGF therapy in terms of IOP change. Pooled analysis revealed that Ozurdex therapy did not lead to a higher rate of severe ocular adverse events than anti-VEGF therapy. Overall, Ozurdex therapy was not inferior to anti-VEGF therapy in terms of VA improvement in patients with nonresistant DME. Although the number of studies was limited, the pooled results revealed that Ozurdex therapy was superior to anti-VEGF therapy in patients with resistant DME. Regarding anatomical outcomes, Ozurdex therapy was superior to anti-VEGF therapy in patients with nonresistant DME as well as in those with resistant DME.

For determining the factors associated with the efficacy of Ozurdex and anti-VEGF therapies, meta-regression was performed on the mean age, treatment duration, baseline BCVA, baseline CRT, HbA1C level, and lens status in patients with nonresistant DME. However, no association was noted between these factors and BCVA change in the linear model. In the nonlinear meta-regression model, an association was noted between the MD in BCVA change and the MD in CRT change when comparing Ozurdex therapy and anti-VEGF therapy. Moreover, an association was noted between the MD in CRT change and CRT. As revealed by the parabola figure, Ozurdex therapy tended to be superior to anti-VEGF therapy if the baseline CRT was more than 410 µm. We thought this result may come from the disease severity. To our knowledge, CRT was the index of the disease severity of DME. In other words, Ozurdex therapy tended to be superior to anti-VEGF therapy in patients with moderate to severe DME. The other possible explanation was the role of inflammatory cytokines and chemokines in the pathogenesis of DME. The severity of the DME may be associated with the number of inflammatory factors. Therefore, Ozurdex therapy, which suppressed the inflammatory cytokines, may be superior to anti-VEGF therapy in patients with moderate to severe DME. Furthermore, Subfoveal serous retinal detachment (SRD), which was a potential factor affecting the efficacy of Anti-VEGF and Ozurdex treatment, was associated with high CRT. This novel finding suggests that clinicians can choose a combination therapy of Ozurdex + anti-VEGF or Ozurdex monotherapy depending on the baseline CRT. Furthermore, crucial factors, including biomarkers of spectral domain optical coherence tomography (SD-OCT) and cytokines in the vitreous body, were analyzed in our study. Unfortunately, sufficient data were not available from the head-to-head studies comparing Ozurdex therapy and anti-VEGF therapy.

In the subgroup analysis of the treatment modality of Ozurdex (combination therapy or monotherapy) on nonresistant DME, the test of the subgroup difference showed no significant difference between combination therapy and monotherapy regarding BCVA change and CRT decrease. However, there was still high heterogeneity in the subgroup analysis. The subgroup analysis of the type of Anti-VEGF was performed to figure out the source of heterogeneity. Regarding the BCVA change, we detected the significant subgroup difference (p < 0.01) between Aflibercept, Ranibizumab, and Bevacizumab. The Aflibercept therapy contributed to more BCVA change (Supplementary-15). However, no significant difference was noted between Aflibercept, Ranibizumab, and Bevacizumab regarding the CRT decrease (p = 0.14) (Supplementary-16). In addition, the subgroup analysis of study design (RCT or observational study) revealed no significant difference between RCT with observational study regarding BCVA change (p = 0.71) and CRT decrease (p = 0.06). (Supplementary-17, 18).

### OCT biomarkers for predicting efficacy

In recent years, researchers have been interested in the potential factors affecting the efficacy of Ozurdex and anti-VEGF therapies. The biomarkers of SD-OCT are considered to be an important factor affecting the efficacy of these two therapies. We summarized the OCT biomarkers which were reported in the included study. (Supplementary-19) Hyperreflective dots (HRDs) are circumscribed dots with a reflective signal higher than or equal to the retinal pigment epithelium. Their morphological characteristics were first described by Bolz et al. and De Benedetto et al.^60,61^. Among the head-to-head studies comparing Ozurdex therapy and anti-VEGF therapy, 5 studies reported outcomes related to HRDs^18,23,28,30,58^. Ceravolo et al.^28^ reported the reduction of HRDs was significantly greater in Ozurdex group. Moreover, Hernández-Bel et al.^23^ reported a significant decrease in HRDs in combination therapy group and anti-VEGF group, with earlier improvements being noted in combination therapy group. In 2017, Vujosevic et al.^18^ reported that both Ozurdex therapy and anti-VEGF therapy could significantly decrease HRDs. They suggested that the number of HRDs at baseline could be a prognostic factor for functional outcomes. In a retrospective study by Vujosevic et al.^30^, Ozurdex therapy resulted in a greater decrease compared with anti-VEGF therapy. Furthermore, they reported that Ozurdex therapy seemed to be more effective in cases with a higher number of HRDs at baseline. In a prospective study by Rübsam et al.^58^, a significant decrease in HRDs was noted only in Ozurdex group and not in anti-VEGF group. Overall, the existing evidence indicates that HRDs may be a potential factor affecting treatment responses in patients with DME. Noteworthily, the existing studies have limitations in terms of a relatively shorter follow-up time and nonrandomized study design. Further research is warranted to determine whether HRDs could serve as a biomarker and give clinicians insights into choosing the optimal treatment.

Subfoveal serous retinal detachment (SRD) is an OCT finding that involves the accumulation of subretinal fluid and a detached retina with a hyperreflective signal^18,62^. To the best of our knowledge, SRD is associated with a higher concentration of cytokines in the vitreous body and is a sign of poor prognosis^63^. Five of our included studies reported the outcomes of SRD. Bolukbasi et al.^21^, Ceravolo et al.^28^, and Demircan et al.^19^ reported that the SRD height significantly decreased after Ozurdex therapy and anti-VEGF therapy, with Ozurdex therapy being superior to anti-VEGF therapy. However, two studies by Vujosevic et al.^18,30^ revealed no significant difference between Ozurdex therapy and anti-VEGF therapy in terms of the change in the SRD height and the rate of SRD resolution. Furthermore, Bolukbasi et al.^21^ reported that Ozurdex therapy decreased CRT to a greater extent than anti-VEGF therapy in patients with SRD. However, no correlation analysis of SRD and functional outcomes was performed in these studies.

Choroidal thickness (CT) is thought to be associated with the severity of diabetic retinopathy. Diabetic retinopathy leads to an increase in the VEGF level, thereby increasing the CT^64^. Among the studies included in our analysis, those by Aksoy et al.^26^ and Vujosevic et al.^30^ reported that both Ozurdex therapy and anti-VEGF therapy significantly decreased the CT. However, Ceravolo et al.^28^ only reported a significant decrease in the CT in Ozurdex group. Disorganization of the retinal inner layers (DRIL) could be an important prognostic factor for visual outcomes in patients with DME^65^. Vujosevic et al.^30^ found that Ozurdex therapy decreased the extension of DRIL more significantly than anti-VEGF therapy. Hence, evidence to prove the correlation of changes in the extension of DRIL with visual outcomes after treatment is still limited.

### Comparison with recent meta-analyses

A previous meta-analysis of Ozurdex therapy versus anti-VEGF therapy was published in 2018^66^. It only included RCTs and included a total of 4 studies. The pooled results revealed that Ozurdex therapy and anti-VEGF therapy had a similar effect on VA improvement, but the former had a better effect on anatomical outcomes. This result was similar to that of our study. However, their study included a limited number of trials. Recently, Veritti et al.^67^ published a meta-analysis discussing the strategies and treatment options for DME. They included 45,032 eyes from RCTs and observational studies. Their study focused on 10 specific questions. The pooled results revealed no significant difference in terms of VA change in Ozurdex group and each type of anti-VEGF group between the RCTs and observational studies. Regarding the anti-VEGF regimen, the pooled results revealed that the fixed regimen was significantly better than the pro re nata regimen and that the frequency of treatment was significantly associated with the outcome of visual improvement. They further reported that aflibercept was significantly superior to bevacizumab and Ozurdex in both RCTs and observational studies. This finding suggested that the type of anti-VEGF could be a potential factor affecting the treatment outcome. Finally, they reported that some baseline characteristics, such as the patient's age, duration of diabetes, and baseline BCVA, were significantly associated with the visual outcome. This finding was not similar to that of our study, possibly because of the difference in the eligibility criteria of the two studies. Overall, Veritti et al.^67^ critically appraised the existing studies surveying DME management. A recent meta-analysis^68^ included the RCTs to compare the intravitreal steroids including triamcinolone acetonide and Ozurdex. They found the intravitreal steroid resulted in similar BCVA change and lower retinal thickness in DME patients. However, they reported that intraocular pressure-related adverse events were higher in the intravitreal group. In our study, the pooled results showed no significant difference in IOP change between Ozurdex group and anti-VEGF group. The different result may be due to the fact that they included not only Ozurdex but also triamcinolone acetonide. The previous study reported that the triamcinolone acetonide group had 2.4 times higher relative risk in IOP change than Ozurdex group. Compared with their study, we focused on the head-to-head analyses of Ozurdex therapy versus anti-VEGF therapy. We tried to answer the crucial clinical question of choosing the optimal intravitreal medication between Ozurdex and anti-VEGF. The nonlinear meta-regression showed the baseline CRT could be a critical factor affecting the anatomical outcome when treating nonresistant DME. We suggested clinicians chose Ozurdex-related therapy when the patient had a baseline CRT of more than 410 µm. In addition, clinicians should consider the predictive OCT biomarker to select the treatment.

### Limitations

Our study has some limitations. First, to analyze the potential factors affecting the effect of anti-VEGF therapy and Ozurdex therapy, we included both RCTs and non-RCTs. The included studies presented heterogeneity in terms of the population, follow-up duration, treatment modality, and trial design. Although we performed a subgroup analysis of the treatment modality, the heterogeneity remained high. Second, to the best of our knowledge, OCT biomarkers and inflammatory biomarkers are crucial factors affecting the effect of Ozurdex and anti-VEGF therapies in patients with DME. However, we could not perform a meta-analysis because of the limited data from the included trials. Therefore, we summarized the findings of the recruited trials. Third, because of the limited number of studies that recruited patients with resistant DME, we could not perform meta-regression to assess patients with resistant DME. Finally, the outcome measures of VA and CRT varied in the included studies. Some studies used Snellen charts for VA measurement, while others used ETDRS charts for VA measurement. Moreover, CRT measurements reported in these studies had different ranges.

## Conclusion

This systematic review and meta-analysis compared the efficacy and safety profile of Ozurdex and anti-VEGF therapies and determined the potential factors contributing to the therapeutic effects. Overall, in patients with resistant DME, Ozurdex therapy was superior to anti-VEGF therapy regarding BCVA change and CMT decrease. In patients with nonresistant DME, Ozurdex therapy was not inferior to anti-VEGF therapy in terms of VA improvement and CRT decrease. The meta-regression of patients with nonresistant DME revealed a novel finding: Ozurdex therapy tends to decrease CRT to a greater extent than anti-VEGF therapy if the baseline CRT is more than 410 µm. Several studies suggested that Ozurdex therapy was superior in DME patients with HRD and SRD. Nevertheless, further high-quality trials are warranted to discuss other issues, such as OCT and inflammatory biomarkers, in DME treatment.

## Supplementary Information

Supplementary Information.

## Data Availability

The datasets used and analyzed during the current study are available from the corresponding author on reasonable request.
