# Supplementary material for: Efficacy and safety profile of intravitreal dexamethasone implant versus antivascular endothelial growth factor treatment in diabetic macular edema: a systematic review and meta-analysis
Source: Sci Rep. 2023 May 8;13:7428. doi: 10.1038/s41598-023-34673-z (PMC10167345; doi:10.1038/s41598-023-34673-z)
Supplement: Supplementary file 1 — Supplementary Information. [file 41598_2023_34673_MOESM1_ESM.pdf]

Efficacy and safety profile of intravitreal dexamethasone implant versus antivascular endothelial growth factor treatment: a systematic review and meta-analysis

(Supplemental Materials)

Supplemental Material

Supplemental Material 1. Search strategy

Supplemental Material 2. Funnel plot of BCVA change

Supplemental Material 3. Funnel plot of CRT change

Supplemental Material 4. Funnel plot of IOP change

Supplemental Material 5. Funnel plot of SAE occurrence

.

Supplemental Material 6. BCVA change – subgroup analysis of nonresistant DME patients

Supplemental Material 7. BCVA change – meta-regression

Supplemental Material 8. CRT change – subgroup analysis of nonresistant DME patients

Supplemental Material 9. CRT change – meta-regression

Supplemental Material 10. Forest plot of IOP change and subgroup analysis of nonresistant DME patients

Supplemental Material 11. IOP change – meta-regression

Supplemental Material 12. Severe ocular adverse events and subgroup analysis of nonresistant DME patients

Supplemental Material 13. Risk of bias assessment (ROB)

Supplemental Material 14. Risk of bias assessment ROBINS-1 (BCVA)

Supplemental Material 15. Subgroup analysis - type of Anti-vegf - BCVA change

Supplemental Material 16. Subgroup analysis - type of Anti-vegf - CRT decrease

Supplemental Material 17. Subgroup analysis - study design - BCVA change

Supplemental Material 18. Subgroup analysis - study design - CRT decrease

Supplemental Material 19. Summary of OCT biomarker

Supplemental Material 1

Search strategy

Supplemental Material 1
Search strategy

Primary search steps:

#1 anti- vascular endothelial growth factors
#2 anti-VEGF
#3 ranibizumab
#4 lucentis
#5 aflibercept
#6 eylea
#7 bevacizumab
#8 avastin
#9 #1 OR #2 OR #3 OR #4 OR #5 OR #6 OR #7 OR #8
#10 ozurdex
#11 intravitreal dexamethasone implant
#12 dexamethasone
#13 #10 OR #11 OR #12
#14 diabetic retinopathy
#15 diabetic macular edema
#16 DME
#17 DMO
#18 cystoid macular edema
#19 CME
#20 #16 OR #17 OR #18 OR #19
#21 #9 AND #13 AND #20

Final syntax in PubMed (an example):

((((((((anti- vascular endothelial growth factors) OR (anti-VEGF)) OR (ranibizumab)) OR (lucentis)) OR (aflibercept)) OR (eylea)) OR (bevacizumab)) OR (avastin)) AND (((ozurdex) OR (intravitreal dexamethasone implant)) OR (dexamethasone))) AND ((((DME) OR (DMO)) OR (cystoid macular edema)) OR (CME)) Sort by: Publication Date

Supplemental Material 2

Funnel plot of BCVA change

Supplemental Material 2

Funnel plot of BCVA change

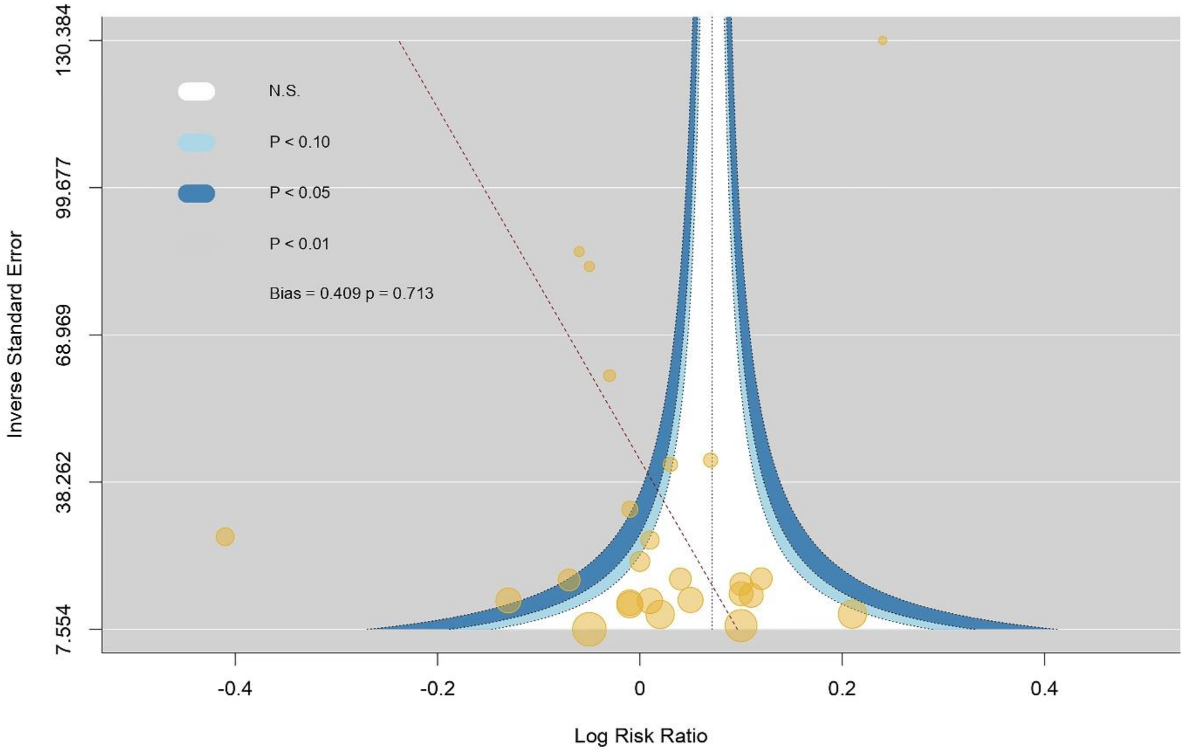

Supplemental Material 3

Funnel plot of CRT change

Supplemental Material 3

Funnel plot of CRT change

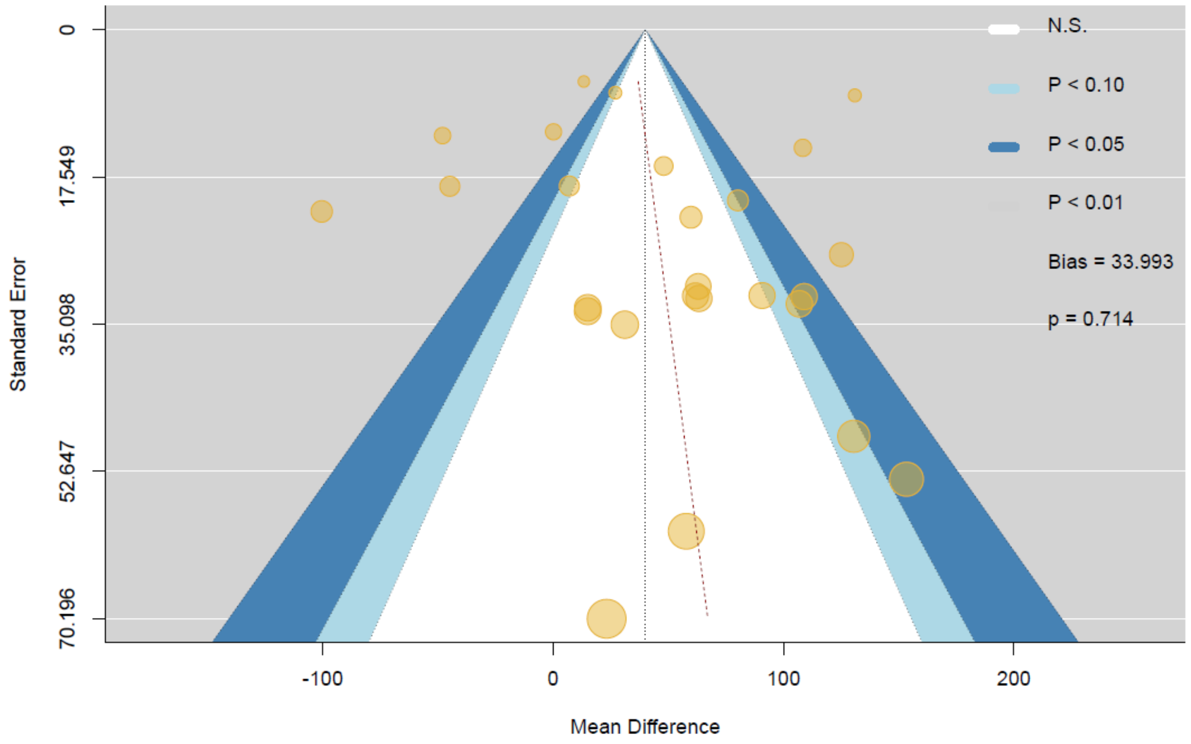

Supplemental Material 4

Funnel plot of IOP change

Supplemental Material 4

Funnel plot of IOP change

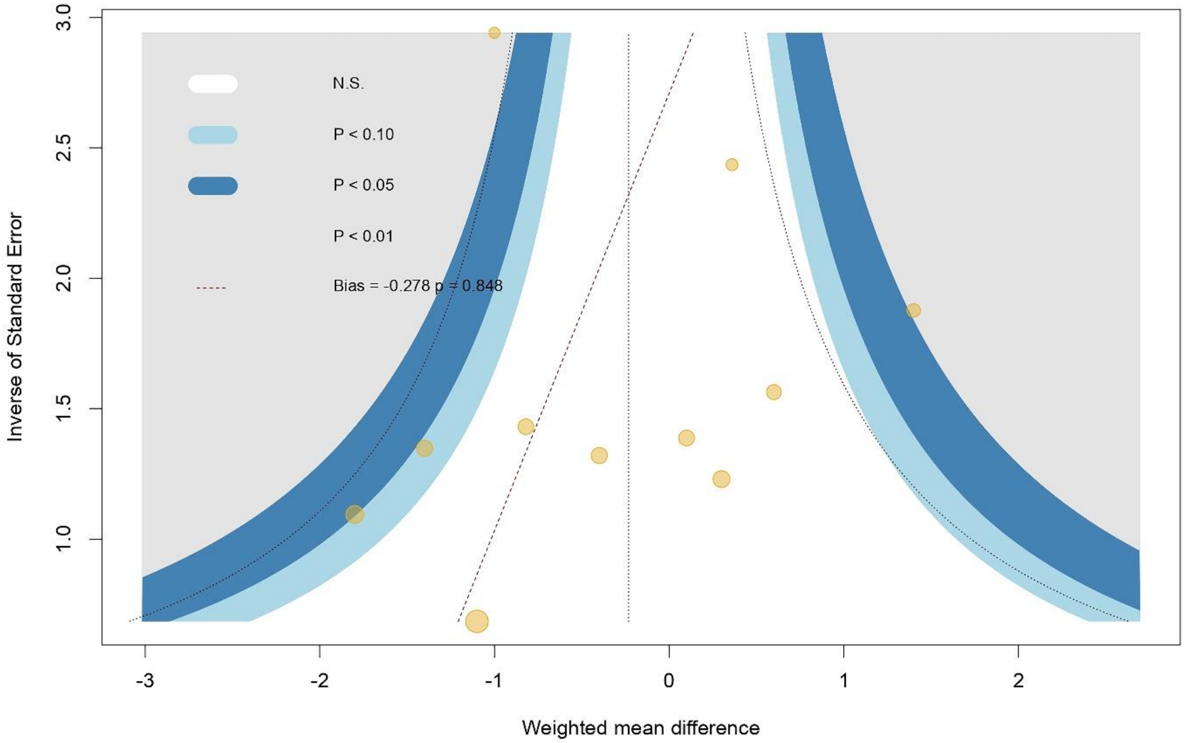

Supplemental Material 5

Funnel plot of SAE occurrence

Supplemental Material 5

Funnel plot of SAE occurrence

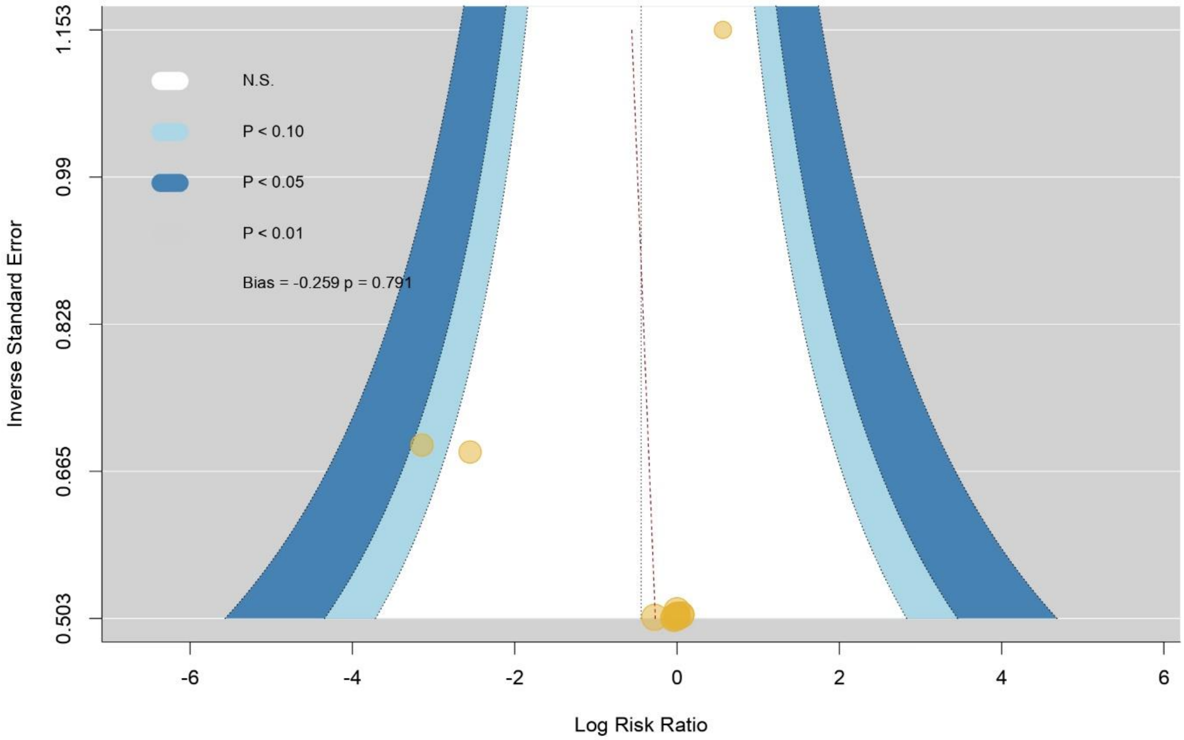

Supplemental Material 6

BCVA change –
subgroup analysis of nonresistant DME patients

Supplemental Material 6

BCVA change – subgroup analysis of nonresistant DME patients

Study	Experimental Mean	Experimental SD	Experimental Total	Control Mean	Control SD	Control Total	MD	95%-CI	Weight
**Subgroup: Anti-VEGF vs Ozurdex alone**									
Aydin	-0.55	0.17	37	-0.14	0.13	28	-0.41	[-0.48; -0.34]	5.1%
Mishra	-0.23	0.32	50	-0.10	0.41	50	-0.13	[-0.27; 0.01]	4.5%
Gilles	-0.18	0.18	42	-0.11	0.33	46	-0.07	[-0.18; 0.04]	4.8%
Ozsaygili	-0.19	0.04	50	-0.13	0.07	48	-0.06	[-0.08; -0.04]	5.3%
Podkowinski	-0.20	0.25	9	-0.15	0.23	5	-0.05	[-0.31; 0.21]	3.3%
Sever	-0.11	0.06	44	-0.06	0.05	40	-0.05	[-0.07; -0.03]	5.3%
Shin	-0.23	0.20	25	-0.22	0.29	20	-0.01	[-0.16; 0.14]	4.4%
Vujosevic	-0.13	0.15	26	-0.13	0.17	23	0.00	[-0.09; 0.09]	5.0%
Shah	-0.11	0.12	23	-0.12	0.15	27	0.01	[-0.06; 0.08]	5.1%
Routier	-0.13	0.05	37	-0.16	0.05	5	0.03	[-0.02; 0.08]	5.2%
Ceravolo	-0.11	0.35	75	-0.15	0.34	81	0.04	[-0.07; 0.15]	4.8%
Comet(L)	-0.14	0.24	19	-0.19	0.22	21	0.05	[-0.09; 0.19]	4.5%
Muftuoglu	-0.09	0.08	18	-0.16	0.06	19	0.07	[0.02; 0.12]	5.2%
Demircan	-0.10	0.30	101	-0.20	0.30	35	0.10	[-0.02; 0.22]	4.7%
Mastropasqua	-0.10	0.35	9	-0.20	0.10	13	0.10	[-0.14; 0.34]	3.5%
Bolubasi	-0.30	0.25	32	-0.40	0.25	25	0.10	[-0.03; 0.23]	4.6%
**Subtotal**			**597**			**486**	**-0.02**	**[-0.09; 0.05]**	**75.4%**
Heterogeneity: $I^2 = 90\%$, $p < 0.01$									
**Subgroup: Anti-VEGF vs Ozurdex+Anti-VEGF**									
Hernández-Bel	-0.25	0.05	15	-0.22	0.04	15	-0.03	[-0.06; 0.00]	5.3%
lin	-0.10	0.34	50	-0.09	0.45	52	-0.01	[-0.16; 0.14]	4.4%
Maturi (2018)	-0.06	0.14	64	-0.05	0.20	63	-0.01	[-0.07; 0.05]	5.2%
Maturi (2015)	-0.10	0.25	19	-0.11	0.21	20	0.01	[-0.14; 0.16]	4.5%
Kaya	-0.19	0.02	34	-0.43	0.04	34	0.24	[0.22; 0.26]	5.3%
**Subtotal**			**182**			**184**	**0.04**	**[-0.10; 0.19]**	**24.6%**
Heterogeneity: $I^2 = 99\%$, $p < 0.01$									
**Overall**			**779**			**670**	**-0.00**	**[-0.06; 0.05]**	**100.0%**
Heterogeneity: $I^2 = 98\%$, $p < 0.01$									
Test for subgroup differences: $\chi^2_1 = 1.19$, df = 1 ($p = 0.27$)									

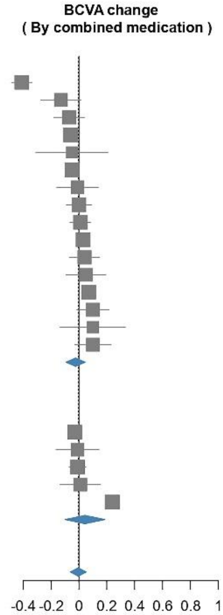

Supplemental Material 7

BCVA change – meta-regression

Supplemental Material 7

BCVA change - meta-regression

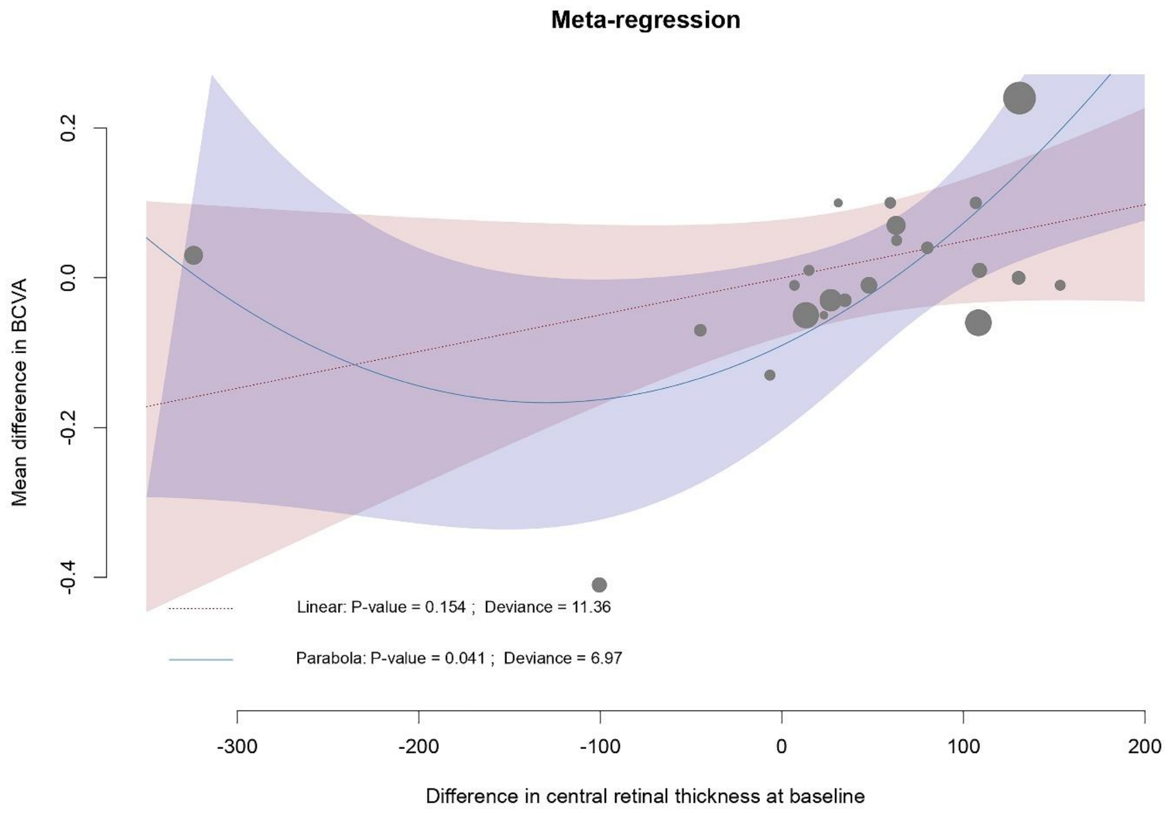

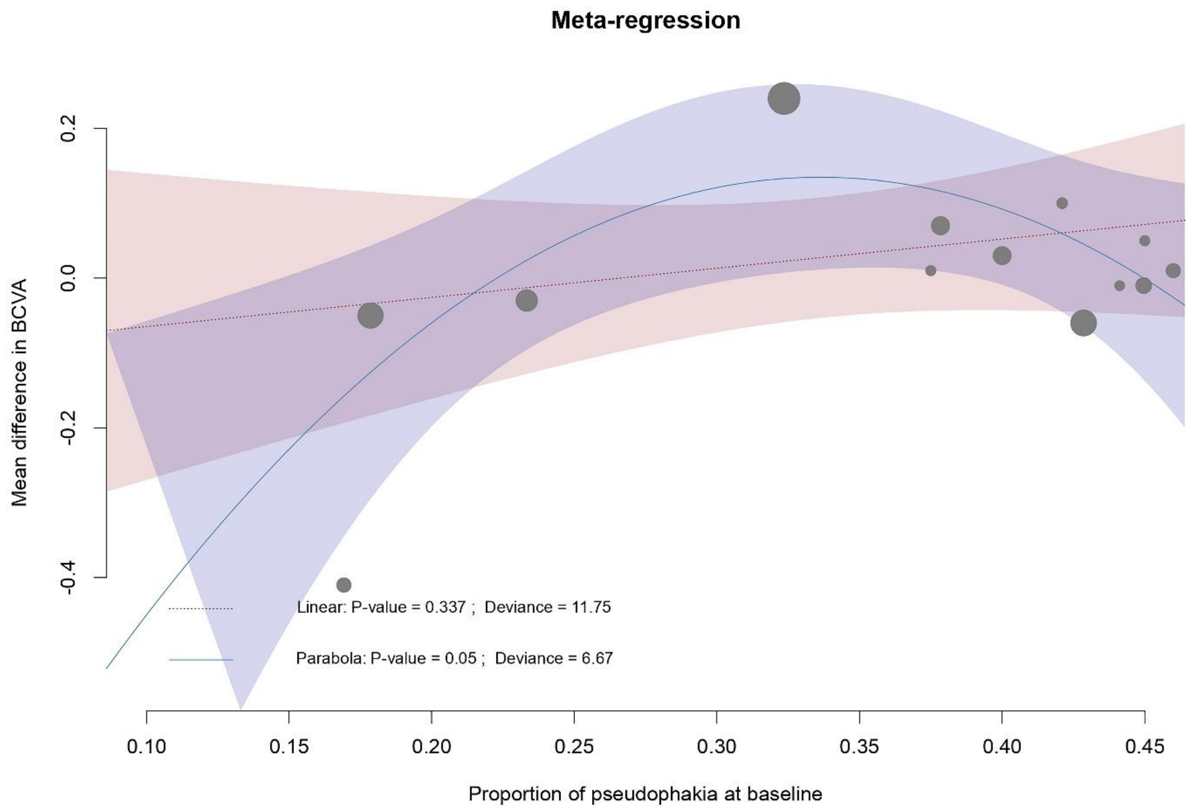

Supplemental Material 8

CRT change –
subgroup analysis of nonresistant DME patients

Supplemental Material 8

CRT change –
subgroup analysis of nonresistant DME patients

Study	Experimental Mean	Experimental SD	Experimental Total	Control Mean	Control SD	Control Total	MD	95%-CI	Weight
**Subgroup: Anti-VEGF vs Ozurdex alone**									
Aydin	-227.10	99.59	37	-126.60	75.14	28	-100.50	[-142.98; -58.02]	5.1%
Routier	-186.00	60.00	37	-138.00	25.00	10	-48.00	[-72.78; -23.22]	5.5%
Gilles	-217.34	100.10	42	-172.50	71.12	46	-44.84	[-81.43; -8.25]	5.2%
Sever	-60.70	27.10	44	-74.00	29.44	40	13.30	[1.16; 25.44]	5.6%
Podkowinski	-77.33	127.77	9	-100.53	124.77	5	23.20	[-114.38; 160.78]	2.5%
Mastropasqua	-179.00	73.60	9	-210.10	90.83	13	31.10	[-37.82; 100.02]	4.3%
Bolubasi	-168.50	82.57	32	-228.30	84.72	25	59.80	[15.97; 103.63]	5.0%
Muftuoglu	-184.00	92.99	18	-247.00	93.05	19	63.00	[3.03; 122.97]	4.6%
Comet(L)	-99.40	92.50	19	-162.70	110.01	21	63.30	[0.50; 126.10]	4.5%
Ceravolo	-168.70	100.57	75	-248.90	150.44	81	80.20	[40.31; 120.09]	5.1%
Demircan	-204.50	176.60	101	-311.40	163.00	35	106.90	[42.85; 170.95]	4.5%
Ozsaygili	-209.10	52.17	50	-317.50	83.23	48	108.40	[80.77; 136.03]	5.4%
Shah	-13.00	105.00	23	-122.00	120.00	27	109.00	[46.63; 171.37]	4.5%
Vujosevic (2020)	-92.37	49.70	18	-217.20	54.47	15	124.83	[88.96; 160.70]	5.2%
Vujosevic (2017)	-163.10	113.40	26	-293.60	206.50	23	130.50	[35.52; 225.48]	3.5%
Shin	-138.50	197.79	25	-291.90	161.60	20	153.40	[48.39; 258.41]	3.3%
**Subtotal**			**565**			**456**	**50.54**	**[11.73; 89.35]**	**73.8%**
Heterogeneity: $I^2 = 92\%$, $p < 0.01$									
**Subgroup: Anti-VEGF vs Ozurdex+Anti-VEGF**									
lin	-109.10	89.86	50	-116.10	98.28	52	7.00	[-29.52; 43.52]	5.2%
Maturi (2015)	-30.00	100.00	19	-45.00	107.00	20	15.00	[-49.97; 79.97]	4.4%
Hernández-Bel	-123.00	19.90	15	-150.00	21.34	15	27.00	[12.23; 41.77]	5.6%
Maturi (2018)	-62.00	97.00	64	-110.00	86.00	63	48.00	[16.13; 79.87]	5.3%
Kaya	-282.00	34.72	34	-413.00	29.78	34	131.00	[115.62; 146.38]	5.6%
**Subtotal**			**182**			**184**	**47.49**	**[-15.93; 110.91]**	**26.2%**
Heterogeneity: $I^2 = 96\%$, $p < 0.01$									
**Overall**			**747**			**640**	**49.58**	**[19.27; 79.89]**	**100.0%**
Heterogeneity: $I^2 = 94\%$, $p < 0.01$									
Test for subgroup differences: $\chi^2_1 = 0.01$, df = 1 ($p = 0.92$)									

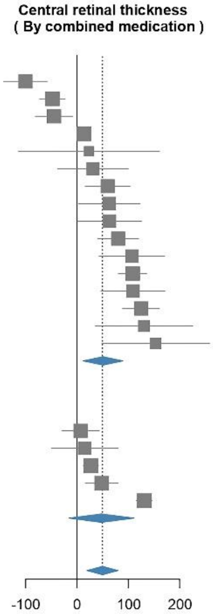

Supplemental Material 9

CRT change – meta-regression

Supplemental Material 9
CRT change –
meta-regression

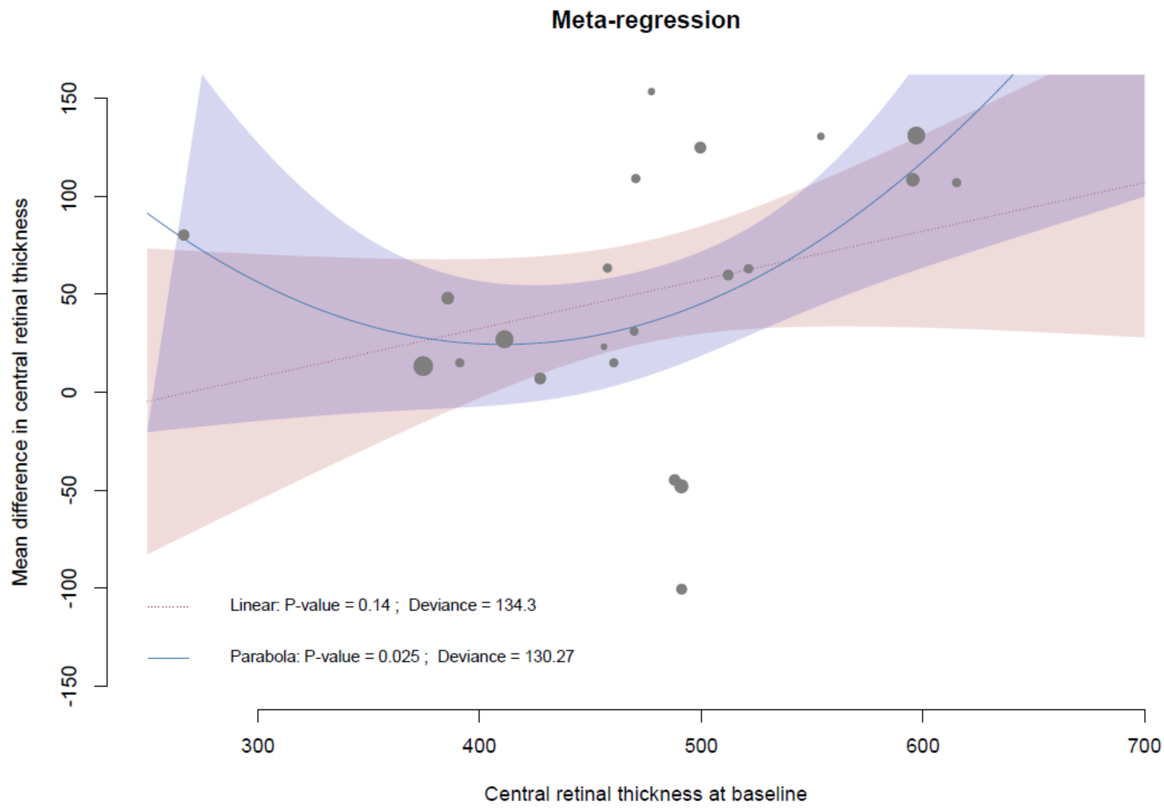

Supplemental Material 10

Forest plot of of IOP change and subgroup analysis of nonresistant DME patients

Supplemental Material 10

Forest plot of IOP change and subgroup analysis of nonresistant DME patients

Study	Experimental Mean	Experimental SD	Experimental Total	Control Mean	Control SD	Control Total	MD	95%-CI	Weight
**Subgroup: non-resistant**									
Shah	-0.70	2.50	23	1.10	3.90	27	-1.80	[-3.59; -0.01]	6.5%
Wickranasingle	-1.30	5.70	22	-0.20	3.80	22	-1.10	[-3.96; 1.76]	3.0%
Aydin	0.80	1.43	37	1.80	1.30	28	-1.00	[-1.67; -0.33]	19.1%
Muftuoglu	-0.45	2.07	18	0.37	2.18	19	-0.82	[-2.19; 0.55]	9.6%
lin	0.00	4.22	50	0.40	3.36	52	-0.40	[-1.88; 1.08]	8.6%
Bolubasi	0.40	2.55	32	0.30	2.81	25	0.10	[-1.31; 1.51]	9.2%
Shin	0.40	2.67	25	0.10	2.74	20	0.30	[-1.29; 1.89]	7.8%
Karakurt(L)	0.08	2.58	81	-0.28	2.16	54	0.36	[-0.44; 1.16]	16.7%
Sever	0.50	3.01	44	-0.10	2.85	40	0.60	[-0.65; 1.85]	10.7%
**Subtotal**			**332**			**287**	**-0.33**	**[-0.92; 0.27]**	**91.2%**
Heterogeneity: $I^2 = 39\%$, $p = 0.11$									
**Subgroup: resistant**									
Sharma	-1.57	2.35	20	-0.17	2.34	20	-1.40	[-2.85; 0.05]	8.8%
**Overall**			**352**			**307**	**-0.42**	**[-1.00; 0.15]**	**100.0%**
Heterogeneity: $I^2 = 40\%$, $p = 0.09$									
Test for subgroup differences: $\chi^2_1 = 1.87$, df = 1 ($p = 0.17$)									

IOP change (By resistant)

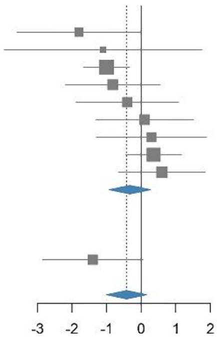

Study	Experimental Mean	Experimental SD	Experimental Total	Control Mean	Control SD	Control Total	MD	95%-CI	Weight
**Subgroup: Anti-VEGF vs Ozurdex alone**									
Shah	-0.70	2.50	23	1.10	3.90	27	-1.80	[-3.59; -0.01]	7.1%
Wickranasingle	-1.30	5.70	22	-0.20	3.80	22	-1.10	[-3.96; 1.76]	3.2%
Aydin	0.80	1.43	37	1.80	1.30	28	-1.00	[-1.67; -0.33]	21.2%
Muftuoglu	-0.45	2.07	18	0.37	2.18	19	-0.82	[-2.19; 0.55]	10.4%
Bolubasi	0.40	2.55	32	0.30	2.81	25	0.10	[-1.31; 1.51]	10.0%
Shin	0.40	2.67	25	0.10	2.74	20	0.30	[-1.29; 1.89]	8.5%
Karakurt(L)	0.08	2.58	81	-0.28	2.16	54	0.36	[-0.44; 1.16]	18.5%
Sever	0.50	3.01	44	-0.10	2.85	40	0.60	[-0.65; 1.85]	11.7%
**Subtotal**			**282**			**235**	**-0.32**	**[-1.01; 0.37]**	**90.6%**
Heterogeneity: $I^2 = 47\%$, $p = 0.07$									
**Subgroup: Anti-VEGF vs Ozurdex+Anti-VEGF**									
lin	0.00	4.22	50	0.40	3.36	52	-0.40	[-1.88; 1.08]	9.4%
**Overall**			**332**			**287**	**-0.33**	**[-0.92; 0.27]**	**100.0%**
Heterogeneity: $I^2 = 39\%$, $p = 0.11$									
Test for subgroup differences: $\chi^2_1 = 0.01$, df = 1 ($p = 0.92$)									

IOP change (By combined medication)

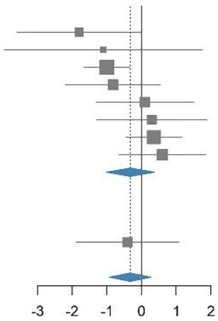

Supplemental Material 11

IOP change – meta-regression

Supplemental Material 11

IOP change – meta-regression

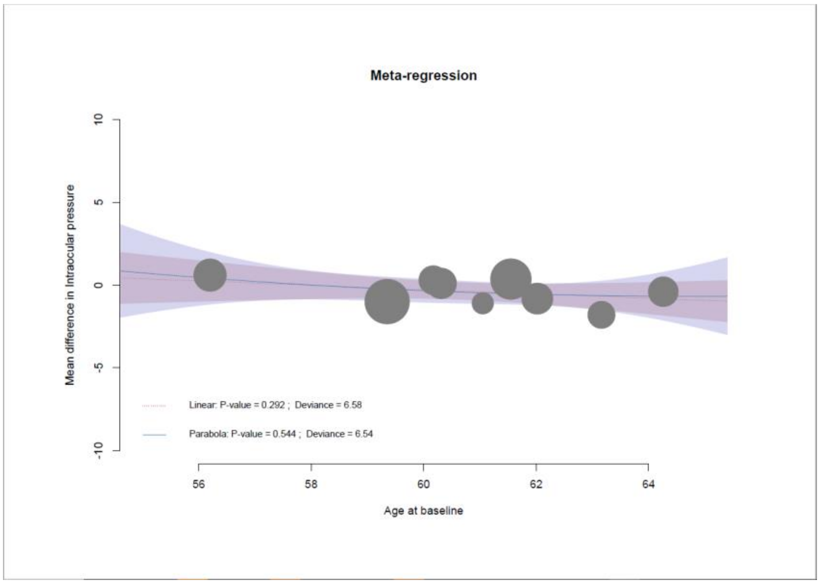

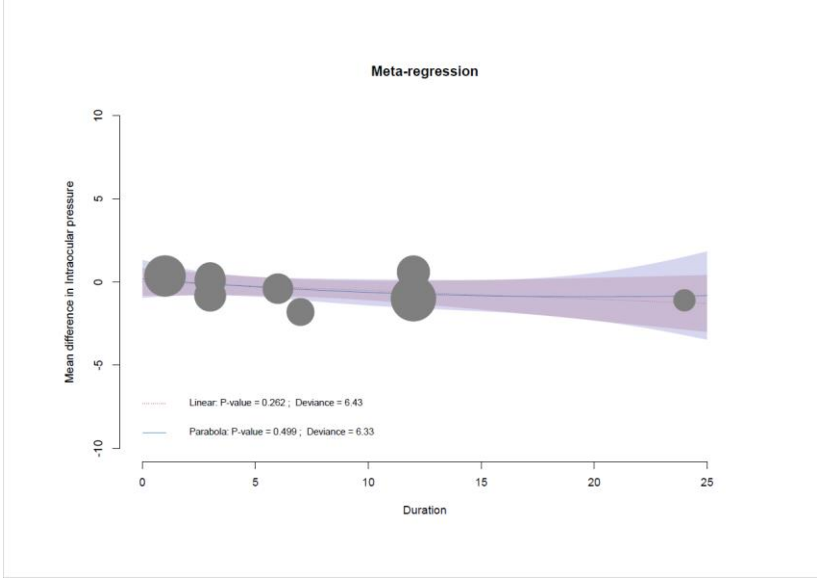

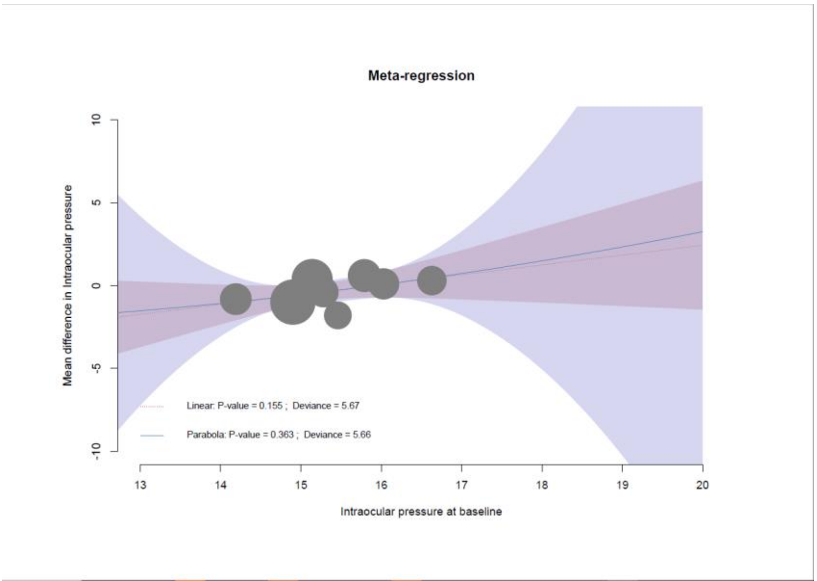

Supplemental Material 12

Severe ocular adverse events and subgroup analysis of nonresistant DME patients

Supplemental Material 12

Severe ocular adverse events and subgroup analysis of nonresistant DME patients

Study	Experimental Events	Experimental Total	Control Events	Control Total	RR	95%-CI	Weight
**Subgroup: non-resistant**							
Callanan	0.0	182.0	11.0	181.0	0.04	[0.00; 0.73]	10.5%
Maturi (2018)	0.0	64.0	6.0	65.0	0.08	[0.00; 1.36]	10.3%
Aydin	0.5	37.5	0.5	28.5	0.76	[0.02; 37.16]	5.5%
Ozsaygili	0.5	50.5	0.5	48.5	0.96	[0.02; 47.45]	5.5%
Kaya	0.5	34.5	0.5	34.5	1.00	[0.02; 48.98]	5.5%
Maturi (2015)	0.5	20.5	0.5	20.5	1.00	[0.02; 48.03]	5.6%
Comet(L)	0.5	20.5	0.5	21.5	1.05	[0.02; 50.43]	5.6%
Muftuoglu	0.5	18.5	0.5	19.5	1.05	[0.02; 50.43]	5.6%
Shah	3.0	23.0	2.0	27.0	1.76	[0.32; 9.64]	29.0%
**Subtotal**		**451.0**		**446.0**	**0.56**	**[0.18; 1.72]**	**83.2%**
Heterogeneity: $I^2 = 8\%$, $p = 0.37$							
**Subgroup: resistant**							
Limon	0.5	30.5	0.5	29.5	0.97	[0.02; 47.17]	5.5%
Sharma	0.5	20.5	0.5	20.5	1.00	[0.02; 48.03]	5.6%
Thomas	0.5	11.5	0.5	11.5	1.00	[0.02; 46.24]	5.7%
**Subtotal**		**62.5**		**61.5**	**0.99**	**[0.94; 1.04]**	**16.8%**
Heterogeneity: $I^2 = 0\%$, $p = 1.00$							
**Overall**		**513.5**		**507.5**	**0.64**	**[0.28; 1.49]**	**100.0%**
Heterogeneity: $I^2 = 0\%$, $p = 0.63$							
Test for subgroup differences: $\chi^2_1 = 1.36$, df = 1 ($p = 0.24$)							

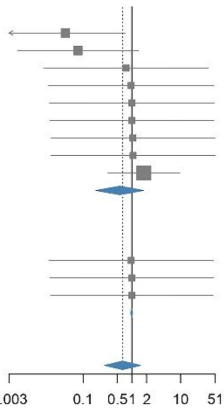

Study	Experimental Events	Experimental Total	Control Events	Control Total	RR	95%-CI	Weight
**Subgroup: Anti-VEGF vs Ozurdex alone**							
Callanan	0.0	182.0	11.0	181.0	0	[0; 1]	13.1%
Aydin	0.5	37.5	0.5	28.5	1	[0; 37]	7.2%
Ozsaygili	0.5	50.5	0.5	48.5	1	[0; 47]	7.2%
Comet(L)	0.5	20.5	0.5	21.5	1	[0; 50]	7.3%
Muftuoglu	0.5	18.5	0.5	19.5	1	[0; 50]	7.3%
Shah	3.0	23.0	2.0	27.0	2	[0; 10]	30.7%
**Subtotal**		**332.0**		**326.0**	**1**	**[0; 3]**	**72.7%**
Heterogeneity: $I^2 = 22\%$, $p = 0.27$							
**Subgroup: Anti-VEGF vs Ozurdex+Anti-VEGF**							
Maturi (2018)	0.0	64.0	6.0	65.0	0	[0; 1]	12.8%
Kaya	0.5	34.5	0.5	34.5	1	[0; 49]	7.2%
Maturi (2015)	0.5	20.5	0.5	20.5	1	[0; 48]	7.3%
**Subtotal**		**119.0**		**120.0**	**0**	**[0; 14]**	**27.3%**
Heterogeneity: $I^2 = 0\%$, $p = 0.42$							
**Overall**		**451.0**		**446.0**	**1**	**[0; 2]**	**100.0%**
Heterogeneity: $I^2 = 8\%$, $p = 0.37$							
Test for subgroup differences: $\chi^2_1 = 0.56$, df = 1 ($p = 0.45$)							

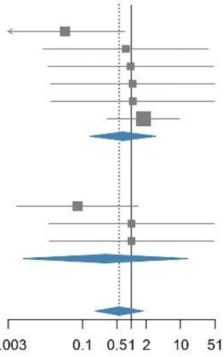

Supplemental Material 13

Risk of bias assessment

ROB

Supplemental Material 13

Risk of bias assessment (ROB)

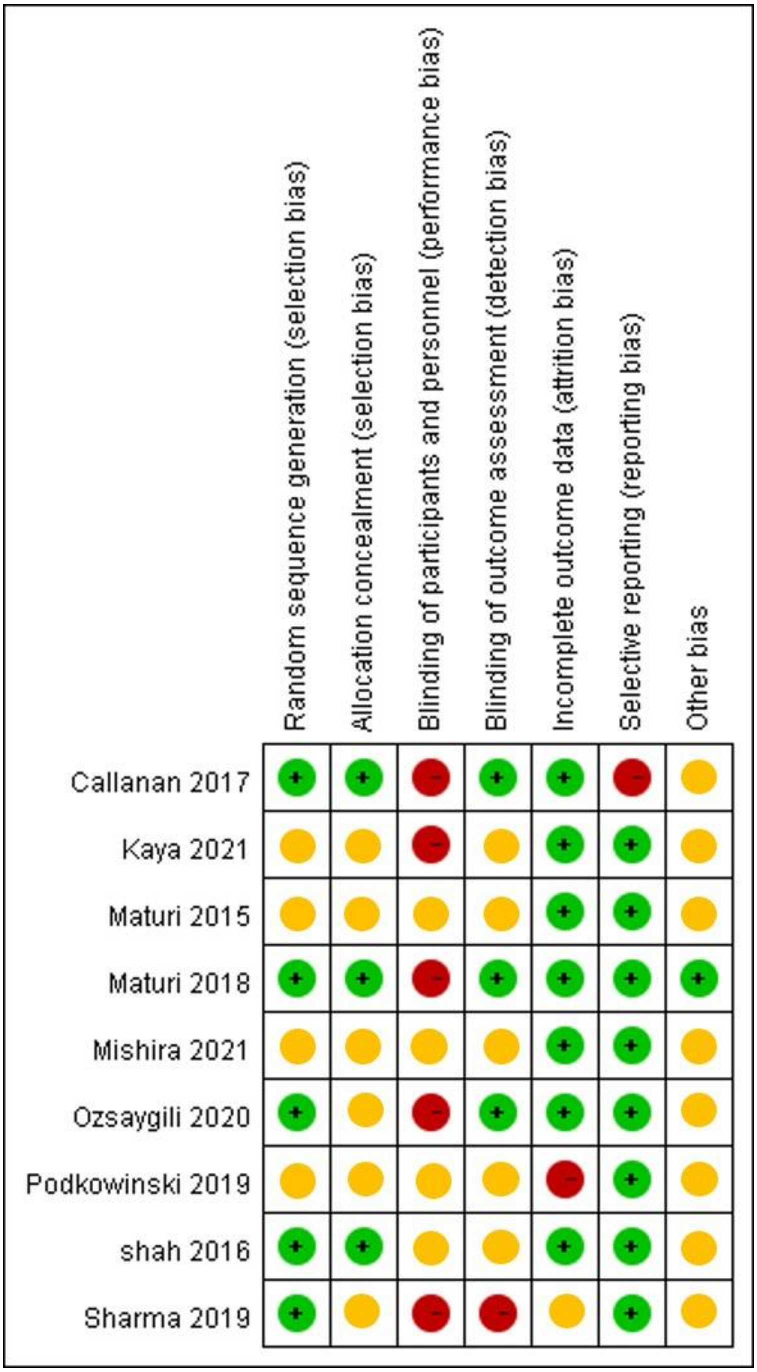

Supplemental Material 14

Risk of bias assessment

ROBINS-1 (BCVA)

Supplemental Material 14

Risk of bias assessment ROBINS-1 (BCVA)

Study	Bias due to confounding	Bias in selection of participants into the study	Bias in classification of interventions	Bias due to deviations from intended interventions	Bias due to missing data	Bias in measurement of outcomes	Bias in selection of the reported result	Overall Bias
AKSOY 2020	Serious	Low	Low	NI	Low	Moderate	Moderate	Serious
Aydin 2020	Low	Low	Low	NI	Low	Moderate	Low	Moderate
Bolubasi 2019	Serious	Low	Low	NI	Low	Moderate	Low	Serious
Busch 2019	Moderate	Low	Low	NI	Low	Low	Low	Moderate
Ceravolo 2020	Low	Low	Low	NI	Low	Moderate	Low	Moderate
Comet 2020	Low	Low	Low	Moderate	Low	Moderate	Low	Moderate
Demircan 2018	Serious	Low	Low	NI	Low	Moderate	Low	Serious
Hernández-Bel 2019	Low	Low	Low	NI	Low	Moderate	Low	Moderate
Limon 2021	Low	Low	Low	Low	Low	Moderate	Low	Moderate
Lin 2021	Serious	Low	Low	NI	Low	Moderate	Low	Serious
Mastropasqua 2019	Low	Low	Low	NI	Moderate	Moderate	Low	Moderate
Muftuogluet 2021	Low	Low	Low	Low	Low	Moderate	Low	Moderate
Routier 2021	Serious	Low	Low	NI	Low	Low	Low	Serious
Rübsam 2021	Serious	Low	Low	NI	Low	Low	Low	Serious
Sever 2017	Serious	Low	Low	NI	Low	Moderate	Low	Serious
Shin 2017	Serious	Low	Low	NI	Moderate	Low	Low	Serious
Thomas 2016	Low	Low	Low	NI	Low	Moderate	Low	Moderate
Vujosevic 2017	Serious	Low	Low	Low	Low	Moderate	Low	Serious

Supplemental Material 15

Subgroup analysis
type of Anti-vegf
BCVA change

Supplemental Material 15

Subgroup analysis
type of Anti-vegf
BCVA change

Study	Experimental Mean	Experimental SD	Total	Control Mean	Control SD	Total	MD	95%-CI	Weight
**Subgroup: L**									
Aydin	-0.55	0.17	37	-0.14	0.13	28	-0.41	[-0.48; -0.34]	5.1%
Mishra	-0.23	0.32	50	-0.10	0.41	50	-0.13	[-0.27; 0.01]	4.5%
Podkowinski	-0.20	0.25	9	-0.15	0.23	5	-0.05	[-0.31; 0.21]	3.3%
Sever	-0.11	0.06	44	-0.06	0.05	40	-0.05	[-0.07; -0.03]	5.3%
Vujosevic (2017)	-0.13	0.15	26	-0.13	0.17	23	0.00	[-0.09; 0.09]	5.0%
Ceravolo	-0.11	0.35	75	-0.15	0.34	81	0.04	[-0.07; 0.15]	4.8%
Comet(L)	-0.14	0.24	19	-0.19	0.22	21	0.05	[-0.09; 0.19]	4.5%
Muftuoglu	-0.09	0.08	18	-0.16	0.06	19	0.07	[0.02; 0.12]	5.2%
Demircan	-0.10	0.30	101	-0.20	0.30	35	0.10	[-0.02; 0.22]	4.7%
Mastropasqua	-0.10	0.35	9	-0.20	0.10	13	0.10	[-0.14; 0.34]	3.5%
Kaya	-0.19	0.02	34	-0.43	0.04	34	0.24	[0.22; 0.26]	5.3%
**Subtotal**			**422**			**349**	**-0.00**	**[-0.12; 0.11]**	**51.2%**
Heterogeneity: $I^2 = 99\%$, $p < 0.01$									
**Subgroup: A**									
Gilles	-0.18	0.18	42	-0.11	0.33	46	-0.07	[-0.18; 0.04]	4.8%
Shin	-0.23	0.20	25	-0.22	0.29	20	-0.01	[-0.16; 0.14]	4.4%
Maturi (2018)	-0.06	0.14	64	-0.05	0.20	63	-0.01	[-0.07; 0.05]	5.2%
Maturi (2015)	-0.10	0.25	19	-0.11	0.21	20	0.01	[-0.14; 0.16]	4.5%
Shah	-0.11	0.12	23	-0.12	0.15	27	0.01	[-0.06; 0.08]	5.1%
Bolubasi	-0.30	0.25	32	-0.40	0.25	25	0.10	[-0.03; 0.23]	4.6%
**Subtotal**			**205**			**201**	**-0.00**	**[-0.05; 0.04]**	**28.5%**
Heterogeneity: $I^2 = 0\%$, $p = 0.55$									
**Subgroup: E**									
Ozsaygili	-0.19	0.04	50	-0.13	0.07	48	-0.06	[-0.08; -0.04]	5.3%
Hernández-Bel	-0.25	0.05	15	-0.22	0.04	15	-0.03	[-0.06; 0.00]	5.3%
lin	-0.10	0.34	50	-0.09	0.45	52	-0.01	[-0.16; 0.14]	4.4%
**Subtotal**			**115**			**115**	**-0.05**	**[-0.09; -0.00]**	**15.0%**
Heterogeneity: $I^2 = 19\%$, $p = 0.29$									
**Subgroup: Non-specified**									
Routier	-0.13	0.05	37	-0.16	0.05	5	0.03	[-0.02; 0.08]	5.2%
**Overall**			**779**			**670**	**-0.00**	**[-0.06; 0.05]**	**100.0%**
Heterogeneity: $I^2 = 98\%$, $p < 0.01$									
Test for subgroup differences: $\chi^2_3 = 11.66$, df = 3 ($p < 0.01$)									

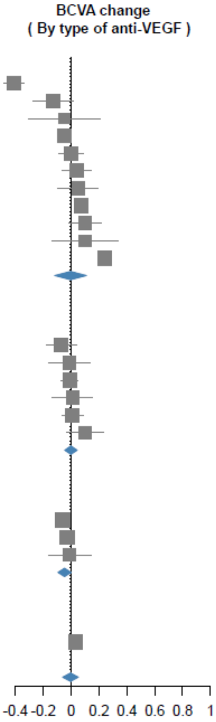

*

Subgroup L= Ranibizumab (Lucentis) group

Subgroup A= Bevacizumab (Avastin) group

Subgroup E= Aflibercept (Eylea) group

Supplemental Material 16

Subgroup analysis
type of Anti-vegf
CRT decrease

Supplemental Material 16

Subgroup analysis
type of Anti-vegf
CRT decrease

Study	Experimental Mean	Experimental SD	Experimental Total	Control Mean	Control SD	Control Total	Central retinal thickness (By type of anti-VEGF)	MD	95%-CI	Weight
**Subgroup: L**										
Aydin	-227.10	99.59	37	-126.60	75.14	28		-100.50	[-142.98; -58.02]	4.9%
Sever	-60.70	27.10	44	-74.00	29.44	40		13.30	[1.16; 25.44]	5.4%
Podkowinski	-77.33	127.77	9	-100.53	124.77	5		23.20	[-114.38; 160.78]	2.4%
Mastropasqua	-179.00	73.60	9	-210.10	90.83	13		31.10	[-37.82; 100.02]	4.1%
Muftuoglu	-184.00	92.99	18	-247.00	93.05	19		63.00	[3.03; 122.97]	4.4%
Comet(L)	-99.40	92.50	19	-162.70	110.01	21		63.30	[0.50; 126.10]	4.3%
Ceravolo	-168.70	100.57	75	-248.90	150.44	81		80.20	[40.31; 120.09]	4.9%
Demircan	-204.50	176.60	101	-311.40	163.00	35		106.90	[42.85; 170.95]	4.3%
Vujosevic (2020)	-92.37	49.70	18	-217.20	54.47	15		124.83	[88.96; 160.70]	5.0%
Vujosevic (2017)	-163.10	113.40	26	-293.60	206.50	23		130.50	[35.52; 225.48]	3.4%
Kaya	-282.00	34.72	34	-413.00	29.78	34		131.00	[115.62; 146.38]	5.4%
**Subtotal**			**390**			**314**		**60.74**	**[13.56; 107.92]**	**48.4%**
Heterogeneity: $I^2 = 95\%$, $p < 0.01$										
**Subgroup: A**										
Gilles	-217.34	100.10	42	-172.50	71.12	46		-44.84	[-81.43; -8.25]	5.0%
Maturi (2015)	-30.00	100.00	19	-45.00	107.00	20		15.00	[-49.97; 79.97]	4.2%
Maturi (2018)	-62.00	97.00	64	-110.00	86.00	63		48.00	[16.13; 79.87]	5.1%
Bolubasi	-168.50	82.57	32	-228.30	84.72	25		59.80	[15.97; 103.63]	4.8%
Shah	-13.00	105.00	23	-122.00	120.00	27		109.00	[46.63; 171.37]	4.3%
Shin	-138.50	197.79	25	-291.90	161.60	20		153.40	[48.39; 258.41]	3.1%
**Subtotal**			**205**			**201**		**48.65**	**[-21.03; 118.34]**	**26.6%**
Heterogeneity: $I^2 = 84\%$, $p < 0.01$										
**Subgroup: E**										
lin	-109.10	89.86	50	-116.10	98.28	52		7.00	[-29.52; 43.52]	5.0%
Hernández-Bel	-123.00	19.90	15	-150.00	21.34	15		27.00	[12.23; 41.77]	5.4%
Ozsaygili	-209.10	52.17	50	-317.50	83.23	48		108.40	[80.77; 136.03]	5.2%
**Subtotal**			**115**			**115**		**47.81**	**[-84.74; 180.36]**	**15.6%**
Heterogeneity: $I^2 = 93\%$, $p < 0.01$										
**Subgroup: Non-specified**										
Routier	-186.00	60.00	37	-138.00	25.00	10		-48.00	[-72.78; -23.22]	5.2%
Rübsam	-74.70	131.24	32	-89.70	105.70	19		15.00	[-50.78; 80.78]	4.2%
**Subtotal**			**69**			[illegible]		**-24.17**	**[-412.37; 364.03]**	**9.5%**
Heterogeneity: $I^2 = 68\%$, $p = 0.08$										
**Overall**			**779**			**659**		**48.10**	**[19.06; 77.13]**	**100.0%**
Heterogeneity: $I^2 = 94\%$, $p < 0.01$										
Test for subgroup differences: $\chi^2_3 = 5.54$, df = 3 ($p = 0.14$)										

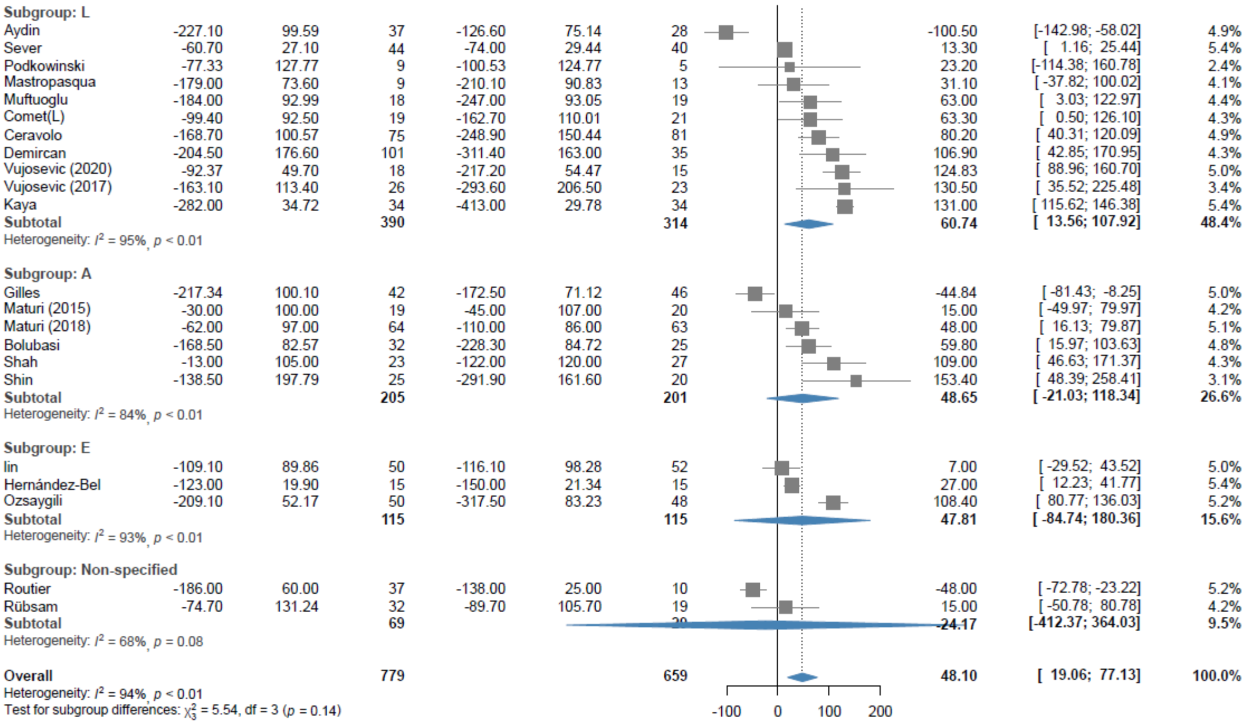

*

Subgroup L= Ranibizumab (Lucentis) group

Subgroup A= Bevacizumab (Avastin) group

Subgroup E= Aflibercept (Eylea) group

Supplemental Material 17

Subgroup analysis
study design
BCVA change

Supplemental Material 17

Subgroup analysis
study design
BCVA change

Study	Experimental Mean	Experimental SD	Total	Control Mean	Control SD	Total	MD	95%-CI	Weight
**Subgroup: Not RCT**									
Aydin	-0.55	0.17	37	-0.14	0.13	28	-0.41	[-0.48; -0.34]	5.1%
Gilles	-0.18	0.18	42	-0.11	0.33	46	-0.07	[-0.18; 0.04]	4.8%
Sever	-0.11	0.06	44	-0.06	0.05	40	-0.05	[-0.07; -0.03]	5.3%
Hernández-Bel	-0.25	0.05	15	-0.22	0.04	15	-0.03	[-0.06; 0.00]	5.3%
Shin	-0.23	0.20	25	-0.22	0.29	20	-0.01	[-0.16; 0.14]	4.4%
lin	-0.10	0.34	50	-0.09	0.45	52	-0.01	[-0.16; 0.14]	4.4%
Vujosevic (2017)	-0.13	0.15	26	-0.13	0.17	23	0.00	[-0.09; 0.09]	5.0%
Routier	-0.13	0.05	37	-0.16	0.05	5	0.03	[-0.02; 0.08]	5.2%
Ceravolo	-0.11	0.35	75	-0.15	0.34	81	0.04	[-0.07; 0.15]	4.8%
Comet(L)	-0.14	0.24	19	-0.19	0.22	21	0.05	[-0.09; 0.19]	4.5%
Muftuoglu	-0.09	0.08	18	-0.16	0.06	19	0.07	[0.02; 0.12]	5.2%
Demircan	-0.10	0.30	101	-0.20	0.30	35	0.10	[-0.02; 0.22]	4.7%
Mastropasqua	-0.10	0.35	9	-0.20	0.10	13	0.10	[-0.14; 0.34]	3.5%
Bolubasi	-0.30	0.25	32	-0.40	0.25	25	0.10	[-0.03; 0.23]	4.6%
**Subtotal**			**530**			**423**	**-0.01**	**[-0.09; 0.06]**	**66.9%**
Heterogeneity: $I^2 = 91\%$, $p < 0.01$									
**Subgroup: RCT**									
Mishra	-0.23	0.32	50	-0.10	0.41	50	-0.13	[-0.27; 0.01]	4.5%
Ozsaygili	-0.19	0.04	50	-0.13	0.07	48	-0.06	[-0.08; -0.04]	5.3%
Podkowinski	-0.20	0.25	9	-0.15	0.23	5	-0.05	[-0.31; 0.21]	3.3%
Maturi (2018)	-0.06	0.14	64	-0.05	0.20	63	-0.01	[-0.07; 0.05]	5.2%
Maturi (2015)	-0.10	0.25	19	-0.11	0.21	20	0.01	[-0.14; 0.16]	4.5%
Shah	-0.11	0.12	23	-0.12	0.15	27	0.01	[-0.06; 0.08]	5.1%
Kaya	-0.19	0.02	34	-0.43	0.04	34	0.24	[0.22; 0.26]	5.3%
**Subtotal**			**249**			**247**	**0.01**	**[-0.10; 0.12]**	**33.1%**
Heterogeneity: $I^2 = 99\%$, $p < 0.01$									
**Overall**			**779**			**670**	**-0.00**	**[-0.06; 0.05]**	**100.0%**

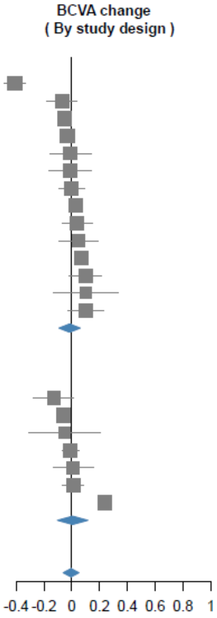

Heterogeneity: $I^2 = 98\%$, $p < 0.01$
Test for subgroup differences: $\chi^2_1 = 0.14$, df = 1 ($p = 0.71$)

Supplemental Material 18

Subgroup analysis
study design
CRT decrease

Supplemental Material 18

Subgroup analysis
study design
CRT decrease

Study	Experimental Mean	Experimental SD	Experimental Total	Control Mean	Control SD	Control Total	MD	95%-CI	Weight
**Subgroup: Not RCT**									
Aydin	-227.10	99.59	37	-126.60	75.14	28	-100.50	[-142.98; -58.02]	4.9%
Routier	-186.00	60.00	37	-138.00	25.00	10	-48.00	[-72.78; -23.22]	5.2%
Gilles	-217.34	100.10	42	-172.50	71.12	46	-44.84	[-81.43; -8.25]	5.0%
lin	-109.10	89.86	50	-116.10	98.28	52	7.00	[-29.52; 43.52]	5.0%
Sever	-60.70	27.10	44	-74.00	29.44	40	13.30	[1.16; 25.44]	5.4%
Rübsam	-74.70	131.24	32	-89.70	105.70	19	15.00	[-50.78; 80.78]	4.2%
Hernández-Bel	-123.00	19.90	15	-150.00	21.34	15	27.00	[12.23; 41.77]	5.4%
Mastropasqua	-179.00	73.60	9	-210.10	90.83	13	31.10	[-37.82; 100.02]	4.1%
Bolubasi	-168.50	82.57	32	-228.30	84.72	25	59.80	[15.97; 103.63]	4.8%
Muftuoglu	-184.00	92.99	18	-247.00	93.05	19	63.00	[3.03; 122.97]	4.4%
Comet(L)	-99.40	92.50	19	-162.70	110.01	21	63.30	[0.50; 126.10]	4.3%
Ceravolo	-168.70	100.57	75	-248.90	150.44	81	80.20	[40.31; 120.09]	4.9%
Demircan	-204.50	176.60	101	-311.40	163.00	35	106.90	[42.85; 170.95]	4.3%
Vujosevic (2020)	-92.37	49.70	18	-217.20	54.47	15	124.83	[88.96; 160.70]	5.0%
Vujosevic (2017)	-163.10	113.40	26	-293.60	206.50	23	130.50	[35.52; 225.48]	3.4%
Shin	-138.50	197.79	25	-291.90	161.60	20	153.40	[48.39; 258.41]	3.1%
**Subtotal**			**580**			**462**	**34.68**	**[-1.50; 70.85]**	**73.4%**
Heterogeneity: $I^2 = 89\%$, $p < 0.01$									
**Subgroup: RCT**									
Maturi (2015)	-30.00	100.00	19	-45.00	107.00	20	15.00	[-49.97; 79.97]	4.2%
Podkowinski	-77.33	127.77	9	-100.53	124.77	5	23.20	[-114.38; 160.78]	2.4%
Maturi (2018)	-62.00	97.00	64	-110.00	86.00	63	48.00	[16.13; 79.87]	5.1%
Ozsaygili	-209.10	52.17	50	-317.50	83.23	48	108.40	[80.77; 136.03]	5.2%
Shah	-13.00	105.00	23	-122.00	120.00	27	109.00	[46.63; 171.37]	4.3%
Kaya	-282.00	34.72	34	-413.00	29.78	34	131.00	[115.62; 146.38]	5.4%
**Subtotal**			**199**			**197**	**82.74**	**[32.67; 132.81]**	**26.6%**
Heterogeneity: $I^2 = 84\%$, $p < 0.01$									
**Overall**			**779**			**659**	**48.10**	**[19.06; 77.13]**	**100.0%**

Heterogeneity: $I^2 = 94\%$, $p < 0.01$
Test for subgroup differences: $\chi^2_1 = 3.46$, df = 1 ($p = 0.06$)

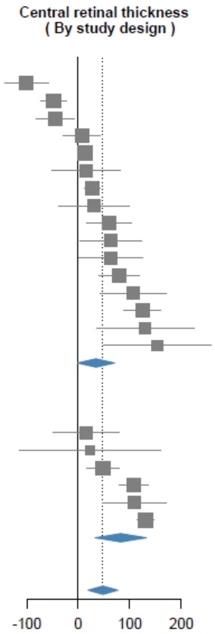

Supplemental Material 19

Summary of OCT biomarker

Supplemental Material 19

Summary of OCT biomarker

Study	Treatment	Hyperreflective dots (HRD) change (numbers)	Hyperreflective dots (HRD) yes/no, after treatment(eye)	Subfoveal serous retinal detachment (SRD) change (um)	Change of choroidal thickness change (um)	disorganization of inner retinal layers (DRIL) (um)
Aksoy et al	1. Aflibercept<br>2. Ozurdex				-92.94<br>-94.58	
Bolubasi et al	1. Bevacizumab<br>2. Ozurdex			-110.69<br>-162.66*		
Ceravolo et al	1. Ranibizumab<br>2. Ozurdex	-6.7<br>-13.22*		-37.4<br>-89.3*	-1.9<br>-28.6	
Demircan et al	1. Ranibizumab<br>2. Ozurdex			-133.6<br>-133.6		
Hernández-Bel et al	1. Aflibercept<br>2. Ozurdex+ Aflibercept		10/5<br>7/8	5/10 (resolution after tx)<br>7/6 (resolution after tx)		
Vujosevic et al 2017	1. Ranibizumab<br>2.Ozurdex	-28<br>-32.4		5/10 (resolution after tx)<br>7/6 (resolution after tx)	-9.3%<br>-11.3%	
Vujosevic et al 2020	1. Ranibizumab<br>2.Ozurdex	-5.94<br>-21.38*		-96.7%<br>-86.8%		-80.52<br>-394.65

* Ozurdex group had significant change comparing with Anti-VEGF group
